# Metaproteomics reveals persistent and phylum-redundant metabolic functional stability in adult human gut microbiomes of Crohn’s remission patients despite temporal variations in microbial taxa, genomes, and proteomes

**DOI:** 10.1186/s40168-019-0631-8

**Published:** 2019-02-11

**Authors:** J. Alfredo Blakeley-Ruiz, Alison R. Erickson, Brandi L. Cantarel, Weili Xiong, Rachel Adams, Janet K. Jansson, Claire M. Fraser, Robert L. Hettich

**Affiliations:** 1 Chemical Sciences Division, Oak Ridge National Laboratory, Oak Ridge, TN 37831 USA; 2 Graduate School of Genome Science & Technology, University of Tennessee, Knoxville, TN 37996 USA; 3 Institute for Genome Sciences, University of Maryland School of Medicine, Baltimore, MD 21201 USA; 4 Department of Medicine, University of Maryland School of Medicine, Baltimore, MD 21201 USA; 5 Biological Sciences Division, Pacific Northwest National Lab, Richland, WA 99352 USA; 6 Current address: U.S. Food and Drug Administration, Center for Food Safety and Applied Nutrition, College Park, MD 20740 USA; 7 Current address: Harvard Medical School, Cell Biology, 240 Longwood Avenue, Boston, MA 02115 USA; 8 Current address: Lyda Hill Department of Bioinformatics, UT Southwestern Medical Center, Dallas, TX 75390 USA

**Keywords:** Gut microbiome, Metaproteomics, Crohn’s disease, Longitudinal analyses, Microbial metabolic function, Human microbiome

## Abstract

**Background:**

The gut microbiome plays a fundamental role in the human host’s overall health by contributing key biological functions such as expanded metabolism and pathogen defense/immune control. In a healthy individual, the gut microbiome co-exists within the human host in a symbiotic, non-inflammatory relationship that enables mutual benefits, such as microbial degradation of indigestible food products into small molecules that the host can utilize, and enhanced pathogen defense. In abnormal conditions, such as Crohn’s disease, this favorable metabolic relationship breaks down and a variety of undesirable activities result, including chronic inflammation and other health-related issues. It has been difficult, however, to elucidate the overall functional characteristics of this relationship because the microbiota can vary substantially in composition for healthy humans and possibly even more in individuals with gut disease conditions such as Crohn’s disease. Overall, this suggests that microbial *membership composition* may not be the best way to characterize a phenotype. Alternatively, it seems to be more informative to examine and characterize the *functional* composition of a gut microbiome. Towards that end, this study examines 25 metaproteomes measured in several Crohn’s disease patients’ post-resection surgery across the course of 1 year, in order to examine persistence of microbial taxa, genes, proteins, and metabolic functional distributions across time in individuals whose microbiome might be more variable due to the gut disease condition.

**Results:**

The measured metaproteomes were highly personalized, with all the temporally-related metaproteomes clustering most closely by individual. In general, the metaproteomes were remarkably distinct between individuals and to a lesser extent within individuals. This prompted a need to characterize the metaproteome at a higher functional level, which was achieved by annotating identified protein groups with KEGG orthologous groups to infer metabolic modules. At this level, similar and redundant metabolic functions across multiple phyla were observed across time and between individuals. Tracking through these various metabolic modules revealed a clear path from carbohydrate, lipid, and amino acid degradation to central metabolism and finally the production of fermentation products.

**Conclusions:**

The human gut metaproteome can vary quite substantially across time and individuals. However, despite substantial intra-individual variation in the metaproteomes, there is a clear persistence of *conserved metabolic functions* across time and individuals. Additionally, the persistence of these core functions is redundant across multiple phyla but is not always observable in the same sample. Finally, the gut microbiome’s metabolism is not driven by a set of discrete linear pathways but a web of interconnected reactions facilitated by a network of enzymes that connect multiple molecules across multiple pathways.

**Electronic supplementary material:**

The online version of this article (10.1186/s40168-019-0631-8) contains supplementary material, which is available to authorized users.

## Background

The human body hosts a dynamic ecosystem of microbial organisms that form an integral part of the overall health maintenance of the host [1]. These microbiota comprise several similar but distinct niches spread out across every surface and cavity of the body, including the skin, nose, mouth, genital track, and gut, where they perform similar but distinct functions [2]. Microbiota, both in terms of population composition and abundances, associated with a healthy human host have been credited with assisting the host with critical functional roles, including expanded metabolism, pathogen defense, immune development, and immune modulation [3–5]. In contrast, microbiota associated with a diseased human host have been associated with unhealthy phenotypes such as obesity, allergies, chronic pain, and inflammation [6–8].

One of the most diverse microbial populations in the human body can be found within the gut [2], where the microbiota play a critical role in assisting the host with metabolism of indigestible food products and immune modulation [5, 9]. Fermentation products produced by the gut microbiota, including short-chain fatty acids, lie at the intersection of human host-microbiota interactions. These fermentation products, particularly butyrate, propionate, and acetate, are a major source of energy for the host, especially in colonocytes [10, 11], and play a role in host immune modulation, health, and disease [3, 12–15].

The composition of the gut microbiota can vary quite substantially across time and individuals. Many factors have been shown to impact this variation, including diet, geography, age, genetic relatedness, and health status [16–18]. Given the number of possible host factors influencing the gut microbiota, it becomes difficult to tease apart what specifically separates the gut microbiota in healthy versus unhealthy individuals. One potential approach to this complicated question is to focus on discrete “metabolic modules” in a gut microbiome, rather than taxa membership or genomic inventory. Many different bacteria share the ability to perform similar metabolic functions. Hence, microbiomes with very different taxonomic and protein compositions could share substantial functional similarity, suggesting that functional redundancy in microbial membership may provide an environmental health advantage [19]. Since proteins are critical participants in the functional activity of life, the direct detection of microbiome-relevant proteins via metaproteomics is ideally suited to help tease apart this problem, and to this end, several studies have demonstrated the ability of LC-MS/MS to measure gut metaproteomes from fecal material, providing a framework for potentially observing gut microbiome function via direct protein detection [18, 20–22].

A few studies have investigated the gut metaproteomes of preterm infants and healthy adults across time, and these studies have revealed variability in both the protein identities as well as overall metabolic activities [23–25]. Recently, the temporal dynamics of the gut microbiome composition in an inflammatory bowel disease cohort that included subjects with Crohn’s disease (CD) having inflammation either in the ileum (ICD) or the colon (CCD), as well as subjects with ulcerative colitis and heathy individuals, were determined by 16S rRNA gene sequencing [26]. All of the subjects with IBD exhibited more volatility in microbiome composition over time, as compared to the healthy individuals, but the degree of dysbiosis from healthy was significantly higher for the ICD patients that had undergone surgery. An open question is how this volatility influences functions carried out by the gut microbiome? To examine this issue, the current study utilizes an integrated metagenomic/metaproteomic approach to investigate the longitudinal stability and variability in the gut metaproteomes of Crohn’s disease patients post resection-surgery. In contrast to previous studies, the focus of this work seeks to go beyond taxa, gene, and protein profiling to investigate microbial metabolic activity at a higher functional level to observe how persistent and redundant function is maintained across varying microbial gut populations. Fecal samples were collected from several adult individuals in remission (post resection surgery) over the course of 1 year (25 samples in total and 5 samples per individual). Metagenomic and metaproteomic data revealed persistent and phylum-redundant metabolic functions despite a significant level of variability in taxa, genes, and proteins.

## Methods

### Patient cohort

This study focused on five human subjects (labeled as P58, P68, P33, P92, and P104), each with a history of Crohn’s disease and resection surgery, which were selected (as detailed below) from a larger Swedish cohort that has been described previously [26]. Five subjects were selected who had multiple fecal samples collected over 1 year. Representatives of both sexes were included in the study (3 females and 2 males), and the subjects were all adults (youngest, born 1967; oldest born 1944). All subjects had undergone resection surgery prior to 2008 and were in remission during this sample collection. (Additional file 1: Table S1).

### Community DNA preparation

Stool (fecal) samples were self-collected and shipped within 1 day to Dr. Jonas Halfvarsson at the Orebro University Hospital in Orebro, Sweden, where they were immediately frozen at − 70 °C upon arrival. The samples were stored frozen until use, and then small portions were excised and thawed immediately prior to DNA extraction to avoid freeze-thaw damage. DNA was extracted from 250 mg of each fecal sample in duplicate. For processing, samples were thawed at 4 °C and, in aliquots of 0.15 g per tube, resuspended in 1 ml of 1 × phosphate-buffered saline. Cell lysis was initiated with two enzymatic incubations, first, using 5 μl of lysozyme (10 mg ml^−1^; Amresco, Solon, OH), 13 μl of mutanolysin (11.7 U μl^−1^; Sigma-Aldrich), and 3 μl of lysostaphin (4.5 U μl^−1^; Sigma-Aldrich) for an incubation of 30 min at 37 °C and, second, using 10 μl proteinase K (20 mg ml^−1^; Research Products International, Mt. Prospect, IL), 50 μl 10% SDS, and 2 μl RNase (10 mg ml^−1^) for an incubation of 45 min at 56 °C. After the enzyme treatments, cells were disrupted by bead beating in tubes with lysing matrix B (0.1-mm silica spheres; MP Biomedicals, Solon, OH), at 6 m s^−1^ for 40 s at room temperature in a FastPrep-24 (MP Biomedicals). The resulting crude lysate was processed using the ZR fecal DNA miniprep kit (Zymo, Irvine, CA) according to the manufacturer’s recommendations. The samples were eluted with 100 μl of ultrapure water into separate tubes. DNA concentrations in the samples were measured using the Quant-iT PicoGreen double-stranded DNA (dsDNA) assay kit (Molecular Probes, Invitrogen, Carlsbad, CA) [27].

### Shotgun metagenomic sequencing and assembly

DNA isolation from stool samples yielded 3–5 μg of purified metagenomic DNA from each of the 15 samples. All metagenomic samples were sequenced using the Illumina platform. Illumina libraries were prepared with the DNA Prep Kit (Illumina, San Diego, CA) following a variation of the manufacturer’s protocol. Following library construction, each sample was subjected to cluster amplification (cBOT) and paired-end sequencing using an Illumina HiSeq2000 according to manufacturer specifications. Raw sequence data were processed using a combination of Illumina RTA/CASAVA software for base-calling and quality scoring and in-house QC pipelines to filter and truncate low-quality reads. Sequences were assembled using NEWBLER by subject after read reduction using khmer [28, 29]. Genes were predicted using MetaGeneMark [30]. Default parameters were used for all assembly related software. Amino acid sequences of all the predicted genes were then compiled into five individual-specific protein databases (each one consisting of a single concatenated, de-replicated metagenome of all three time-points per individual). The metagenome sequence data can be retrieved using the following URL for the NCBI SRA data deposit, under project ID 46321: http://www.ncbi.nlm.nih.gov/sites/entrez?db=bioproject&cmd=Retrieve&dopt=Overview&list_uids=46321

### Metaproteomics sample collection

Each fecal sample for metaproteome measurements (~ 130 mg for each sample) was solubilized in 1 mL SDS lysis buffer (4% w/v SDS, 100 mM Tris·HCl (pH 8.0), 10 mM dithiothreitol (DTT)), sonically disrupted (40% amplitude, 10-s pulse with 10-s rest, 2-min total pulse time), incubated at 95 °C for 5 min, and centrifuged at 21,000 × g. An aliquot of each crude protein extract was quantified using a bicinchoninic acid (BCA)-based protein assay kit (Pierce), and yielded about 4 mg/mL protein for each sample. The crude protein extract was precipitated by trichloroacetic acid (TCA), pelleted by centrifugation, and washed with ice-cold acetone to remove lipids and excess SDS, as described previously [18]. The protein precipitates were resolubilized via sonication in 500 μl of 8 M urea in 100 mM Tris·HCl (pH 8.0) and reduced by incubation with DTT at a final concentration of 10 mM for 1 h at room temperature. Samples were normalized for total protein at this step by using 1 mg of crude protein for each sample, which was then diluted further with 100 mM Tris·HCl and 10 mM CaCl2 (pH 8.0) to a final urea concentration below 4 M. Proteolytic digestions were initiated with sequencing grade trypsin (1/100, w/w; Promega) and incubated overnight at room temperature. A second aliquot of trypsin was added (1/100) after the reactions were diluted with 100 mM Tris·HCl (pH 8.0) to a final urea concentration below 2 M. Following digestion, the peptides were acidified (protonated) in 200 mM NaCl and 0.1% formic acid, filtered with a 10 kDa molecular weight cutoff spin column (Sartorius) to remove under digested proteins. Final peptide solutions were then quantified using bicinchoninic acid (BCA)-based protein assay kit (Pierce) to enable uniform sample injection onto the LC column.

### LC-MS/MS

Peptide mixtures were analyzed in technical replicate measurements via two-dimensional liquid chromatography tandem mass spectrometry (LC/LC-MS/MS) on an LTQ-Orbitrap-Elite mass spectrometer (ThermoFisher Scientific). Peptides (100 µg per sample) were loaded and separated on-line using a bi-phasic 2D (strong-cation exchange (SCX) and C18 reverse phase (RP))-LC column. Each peptide sample was first washed off-line to remove residual urea and NaCl and was then placed in-line and analyzed via 22-h 2D-LC-MS/MS. All samples were analyzed by 11 salt pulses (5%, 7.5%, 10%, 12.5%, 15%, 17.5%, 20%, 25%, 35%, 50%, and 100% of 500 mM ammonium acetate) each followed by a 110 min gradient to 50% solvent B (70% acetonitrile, 30% HPLC grade water, 0.1% formic acid) with the following profile: 0 to 10% solvent B in 10 min, 10 to 35% solvent B in 75 min, and 35 to 50% solvent B in 25 min. Mass spectral data were acquired using Xcalibur in data-dependent acquisition mode for each chromatographic separation. One precursor MS scan was acquired in the Orbitrap at 30 K resolution followed by ten data-dependent MS/MS scans (m/z 400–1700) at 35% normalized collision energy with dynamic exclusion enabled at 1.

### Informatics and quantification

Custom-built FASTA target-decoy databases were generated for each individual by combining that individual’s specific protein database mentioned above with the human genome and common contaminants. The MS raw data for each sample was searched against the individual-specific protein database using MyriMatch/IDPicker with a PSM false discovery rate (FDR) filter of less than 2% [31, 32]. MyriMatch automatically concatenates a reversed database to the forward version prior to searching to enable proper FDR calculation. Protein sequences were clustered into protein groups at ≥ 90% sequence identity using USEARCH (v5.0) [33]. A protein group was classified as identified if it had at least one unique peptide and two distinct peptides, as resolved with in-house scripts. Mass spectra were assigned to protein groups using spectral balancing, as previously described [15, 25, 34].

### Data analysis

The protein databases were functionally annotated with Kyoto Encyclopedia of Genes and Genomes (KEGG) orthologous groups and phylum and genus level taxonomical assignments using Ghost KOALA [35]. Phylum level taxonomy and KEGG orthologous groups were assigned to each protein group using the Ghost KOALA annotations of the seed sequence for each protein group. The KEGG assignments to each identified protein group were further used to infer and quantify human gut microbiome-specific metabolic modules using GOmixer [36, 37]. A metabolic module was inferred if ≥ 33.3% of the enzymatic steps in the module were covered within a single phylum. The phylum abundance of each module was calculated in GOMixer by using the mean spectral count of each phylum’s protein groups that mapped to the module. To examine statistical variance between samples, Spearman correlation coefficients between samples were calculated and hierarchically clustered into a heatmap using Euclidian distance. Technical reproducibility was evaluated using Spearman correlation coefficients between measurements of the same sample. All statistics were calculated using Python scripts. All figures were rendered using Python scripts (https://www.python.org/) or Excel. All figures were refined for quality and sizing in Inkscape (www.inkscape.org). All python scripting was done using the following libraries: Pandas, NumPy, Seaborn (https://seaborn.pydata.org/), Matplotlib, and SciPy [38–41]. Functional influence of each phylum was calculated by taking the total number of KEGG orthologous groups identified with protein evidence in each phylum by sample and dividing that by the total number of KEGG orthologous groups found in the sample’s protein database.

## Results and discussion

### Sequence-guided sample selection and metagenome assembly

16S rRNA sequences were previously generated and published for 135 fecal samples from a Swedish cohort of IBD patients and healthy subjects [26]. From this same sample collection, five individuals with ileal Crohn’s disease (ICD) were selected for deeper analysis of microbial function by metagenomics and metaproteomics because these ICD individuals showed the greatest microbiome volatility and were the most disparate compared to healthy individuals [26].

Shotgun metagenomic sequence data were generated from three fecal samples per each individual, corresponding to an initial time point and at two subsequent 6-month intervals. Metaproteome measurements were conducted on the same samples, plus an additional two intervening time-points for each individual, ensuring that the protein data was adequately represented by metagenomes on either side of the sampling dates.

### Individual gut metaproteomes reveal substantial proteome variability

A total of 14,850 non-redundant protein groups were identified in this study from the 25 samples. Of these protein groups, 494 were human, and 14,356 were microbial. A total of 732–2900 microbial and 119–222 human protein groups were found in each sample (Additional file 1: Figure S1A). Although relatively few human protein groups were identified in total number, they were quite abundant in most samples and comprised a substantial proportion of the assigned spectra. (Additional file 1: Figure S1B). The measurement correlation for all technical replicates had an R^2^ greater than 0.9 and a slope within 0.1 of 1, indicating high technical reproducibility (Additional file 1: Figure S2).

**Fig. 1:**
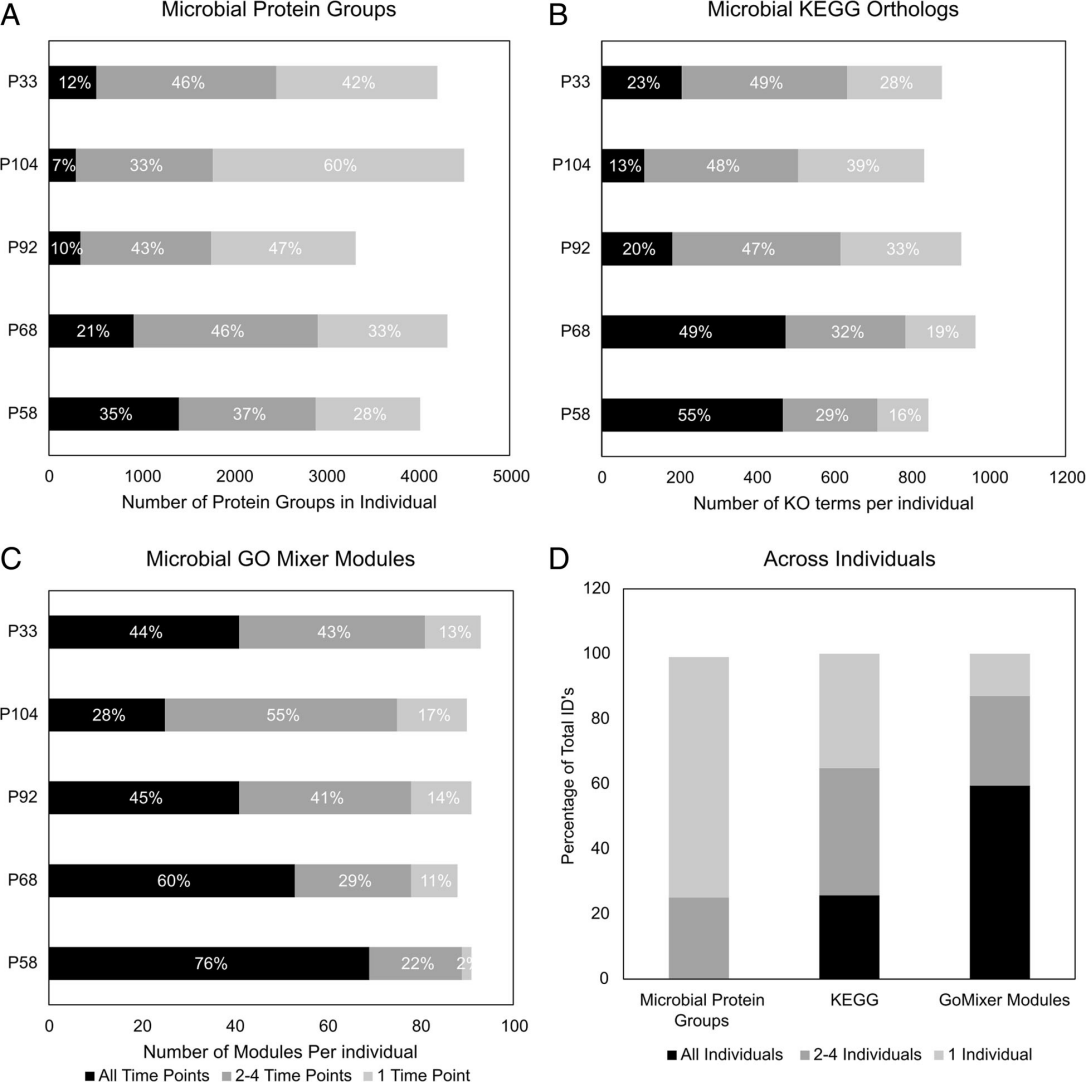
Illustration of how much variation there is in the protein groups identified across time and individuals, and how that variation decreases when resolution is restricted to functional groups. **a** The number of microbial protein groups that were observed across all time points, more than one time point, and only one time point for each individual. **b**, **c** KEGG orthologous groups and GoMixer modules, respectively. **d** The percentage of microbial protein groups, KEGG orthologs, and GoMixer modules that are found across all individuals, more than one individual, and only one individual

As evident by the number of protein groups identified in each sample, qualitative variation between samples was high. Relatively few identical microbial protein groups were observed in all samples (these proteins are further characterized and described in Additional file 1: Appendix S1). Additionally, only 168 microbial protein groups were observed at least once in every individual. Even within a single individual, qualitative variation was high. In individual P104, only 7% of the microbial protein groups identified in that individual were observed across all time points (Fig. 1a). Individuals P33, P104, and P92 had the most varied metaproteomes across time, whereas P58 and P68 had more stable metaproteomes. However, even though individual P58 had the most qualitatively stable metaproteome, less than 36% of its microbial protein groups were found across all time points (Fig. 1a).

**Fig. 2:**
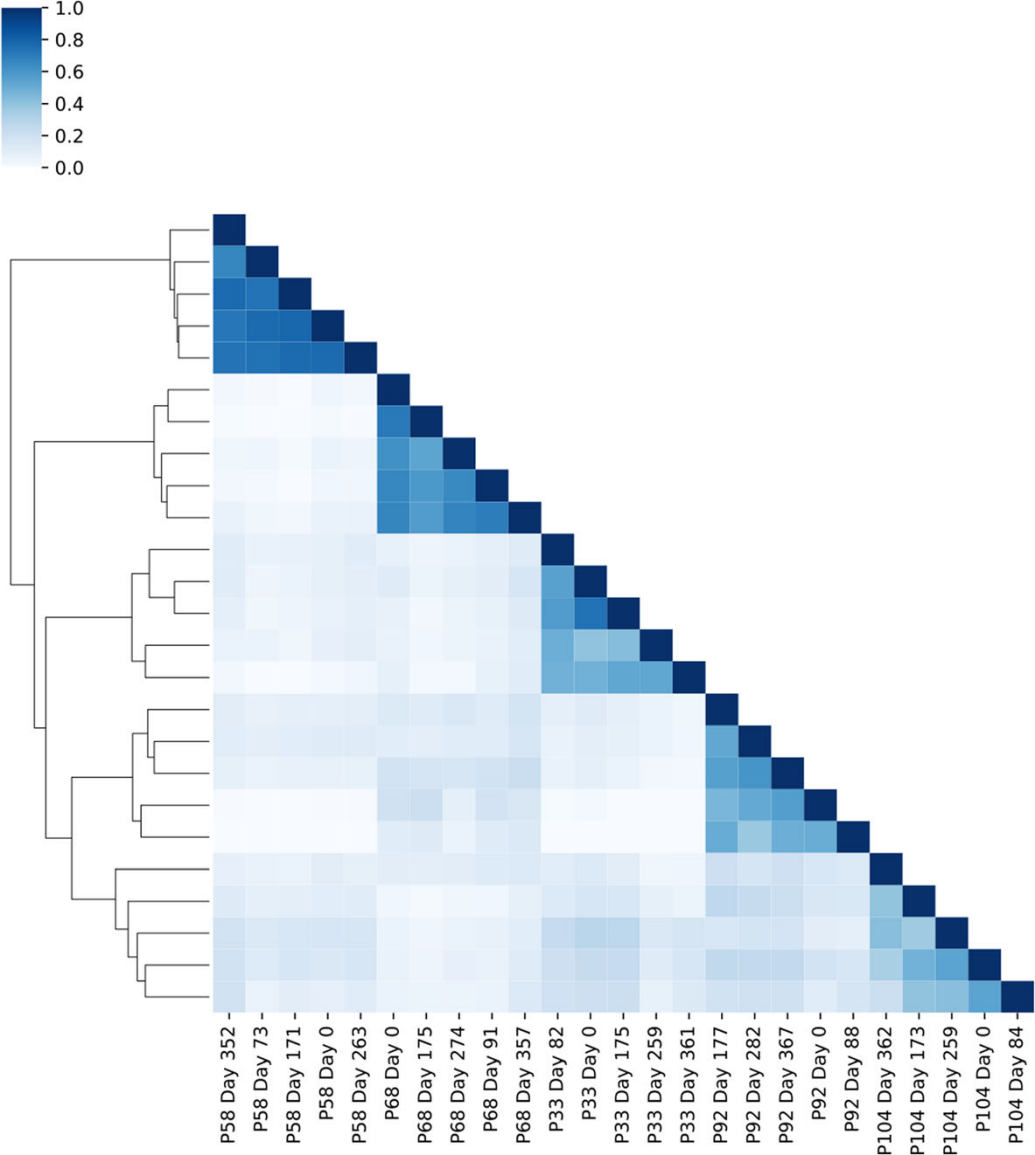
Heatmap illustrating the hierarchical clustering of the Spearman correlation coefficient between samples. Color represents the Spearman correlation coefficient between samples based on protein group abundance. Samples were hierarchically clustered using a Euclidian distance that was calculated based on the correlation coefficients

With this much variation, it was deemed that Spearman correlation coefficients were best suited to quantify the similarity or dissimilarity of the metaproteomes between measurements (Fig. 2). Hierarchical clustering of the correlation between measurements revealed a personalized metaproteome. All measurements clustered more closely to samples from the same individual than samples from any other individual (Fig. 2). These results are similar to a previous study that examined the metaproteomes of healthy adult individuals over time [24]. The idea of personalized gut metaproteomes appears to be a distinct feature of adult metaproteomes, as a recent study of preterm infants showed that the gut metaproteomes of preterm infants did not necessarily cluster by individual over time [25], indicating that individual-specific metaproteomes develop between early infancy and adulthood.

Overall these results suggest that there is an underlying continuity in metaproteomes from the same individual. However, due to the protein variability found within the individuals even with the most similar metaproteomes (P58 and P68), any real observations of functional similarity between samples are likely more meaningful by observing common identifiers of function, such as KEGG orthologous groups, instead of the individual taxa, genes, or proteins.

### Assignment of KEGG orthologous groups reveals remarkable functional redundancy among protein groups

KEGG can be used to infer the functional and taxonomic association of protein groups based on the biochemical function of the orthologous groups (KEGG orthology group (KO) terms) [42] and was employed here to classify and interrogate the metaproteome information. For 99% of all protein groups, KO terms and phylum assignments were unambiguous. For this reason, the annotation of the seed sequence for each protein group was used for the annotation of the entire protein group. The KEGG annotations for each protein sequence and related protein group are detailed in Additional file 2: Spread Sheet 1. The protein groups, their final KEGG annotations, and their abundance per measurement are listed in Additional file 3: Spread Sheet 2.

An average of 165,451 ORF’s per protein database was assigned a KO term. This translated into an average of 5504 predicted KO terms per individual, indicating high functional redundancy across protein sequences. Similarly, this high functional redundancy was also observed across identified protein groups, where 10,172 identified protein groups were assigned a KO term but only amounted to a total of 1573 KO terms. These results are consistent with the concept that many of the different protein groups identified likely have the same function. This is supported by the much more expansive HMP project, which reveals similar available metabolic functions despite varied taxonomy [2].

Within an individual, 16% of the KO terms predicted by each individual’s protein database had protein evidence in at least one of their five samples (Additional file 1**:** Figure S3). For context, less than 2% of the protein groups predicted for each individual had protein evidence in at least one sample. The majority of the observed functions was centered around four phyla: Firmicutes, Bacteroidetes, Actinobacteria, or Proteobacteria. These phyla represented the greatest percentage of the total number of KO terms predicted by each sample’s respective protein database (Additional file 1: Figure S4). Similarly, Firmicutes, Bacteroidetes, Actinobacteria, and Proteobacteria along with Verrucomicrobia were the most represented phyla in previous human gut metaproteomic studies [24, 43]. Although they were observed in this study, Verrucomicrobia did not represent a significant proportion of the identified protein groups. This could be related to the compromised health state of the individuals in the current study as compared to the other studies of healthy individuals. While not as dominant as the other four phyla, protein groups associated with Fusobacteria and Euryarchaeota in P58 and P33, respectively, represented an uncharacteristically high percentage of the total number of KO terms predicted by their associated protein databases (Additional file 1: Figure S4).

Variability between samples decreased when the qualitative analysis was constrained to the KO term level. Between individuals, 25% of the KO terms identified across all samples were seen at least once in every individual. There was also lower intra-individual variability. A greater percentage of the total number of detected KO terms in each individual was also found across time when compared to the protein groups (Fig. 1). These followed essentially the same trend as the protein group level observations, with P104, P33, and P92 being more variable than P58 and P68 (Fig. 1). This suggests two conclusions: the trends in terms of the most variable individuals across time hold true through to the KO term level, and there is overall less qualitative variation between samples when focusing on functional groups instead of protein groups (Fig. 1).

### Inferred metabolic modules demonstrate persistence of metabolic function in a dynamic microbiome

**Fig. 3:**
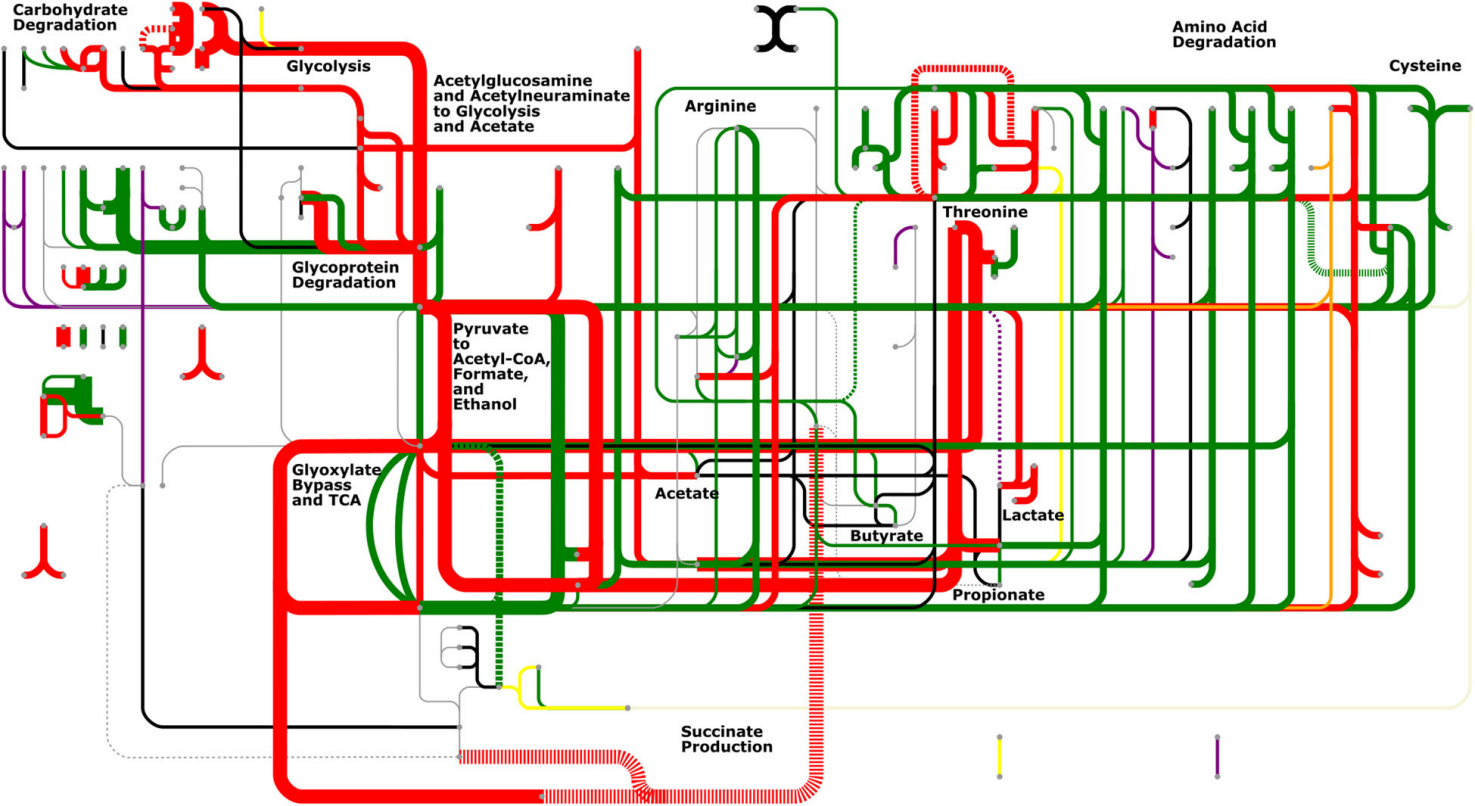
Metabolic map of all the GOMixer modules rendered in the GOMixer web application, http://www.raeslab.org/omixer/visualisation/map. Highlighted lines represent all the modules that were inferred with peptide evidence in at least one sample. Line thickness represents how many samples the module was observed in with the thickest lines representing modules that were represented across all samples, the medium thickest lines being represented by modules inferred in all individuals, and the least thick lines representing modules not seen in all individuals. Red colored lines represent modules that were observed in Firmicutes, Actinobacteria, Bacteroidetes, and Proteobacteria. Green lines represent modules that were observed in at least two of the phyla. Black lines represent modules only represented in Firmicutes. Yellow lines represent modules only observed in Bacteroidetes. Purple lines represent modules only represented in Proteobacteria. Two modules that were unique to other phyla were MF0027 inferred in Euryarcheota and MF0010 inferred in Fusobacteria. These were colored beige and orange respectively

To expand the search for functional similarity between samples, the metabolic dynamics of this metaproteomic dataset were explored using a map of human microbiome derived metabolic modules created by the Raes lab [36], which represent a set of reactions responsible for the conversion of one compound to another, and are inferred via enzyme evidence from the metaproteomic datasets. This metabolic map is available in an online tool called “GOmixer” at http://www.raeslab.org/gomixer/ and is illustrated for this study in Fig. 3. One hundred sixteen out of a total of 133 metabolic modules in “GOmixer” were inferred for specific phyla using the Ghost KOALA annotations for each protein group. A variety of phyla contributed proteomic evidence for these modules; however, in agreement with the trends in Additional file 1: Figure S4, most of the inferred modules were populated with protein groups that originated in Firmicutes, Bacteroidetes, Proteobacteria, or Actinobacteria. Two or more phyla provided evidence for most of the modules observed in Fig. 3, where the green lines indicate modules that have protein evidence in two or more phyla, and the red lines indicate modules that have protein evidence in all four of the main phyla described above. Together, these modules indicate protein evidence for high redundancy in metabolic function between gut microbiome phyla.

**Fig. 4:**
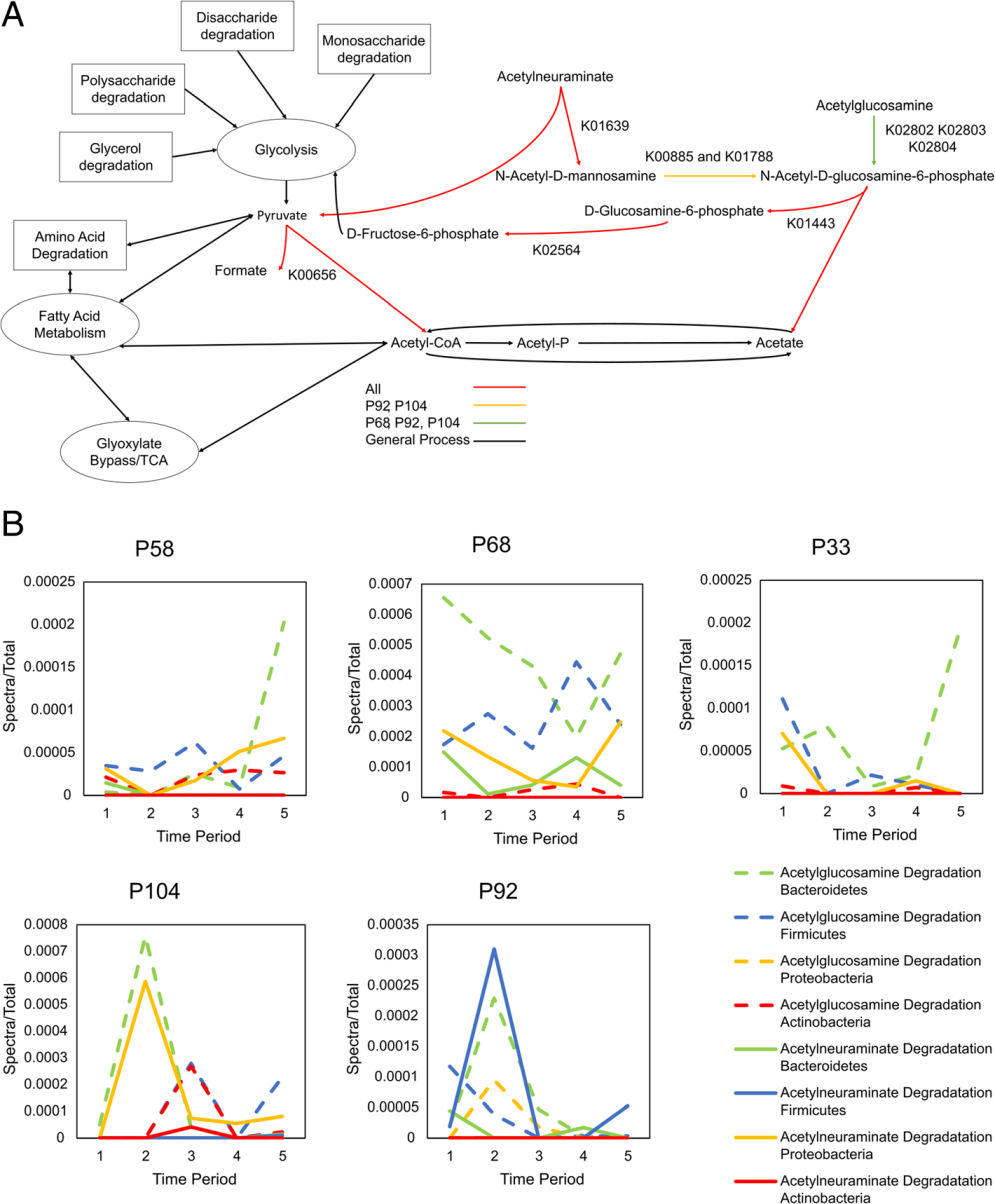
**a** Detailed pathway describing modules MF0003 and MF0005, which together degrade acetylglucosomine and acetylneuraminate into pyruvate, fructose-6-phospate, and acetate. All of the KEGG ortholog terms in this figure had peptide evidence in at least one sample. K00656, while not a part of these modules, but serves as anchor point between these modules and central metabolism. **b** Line graphs that depict the abundance of each module by phylum across time for each individual

**Fig. 5:**
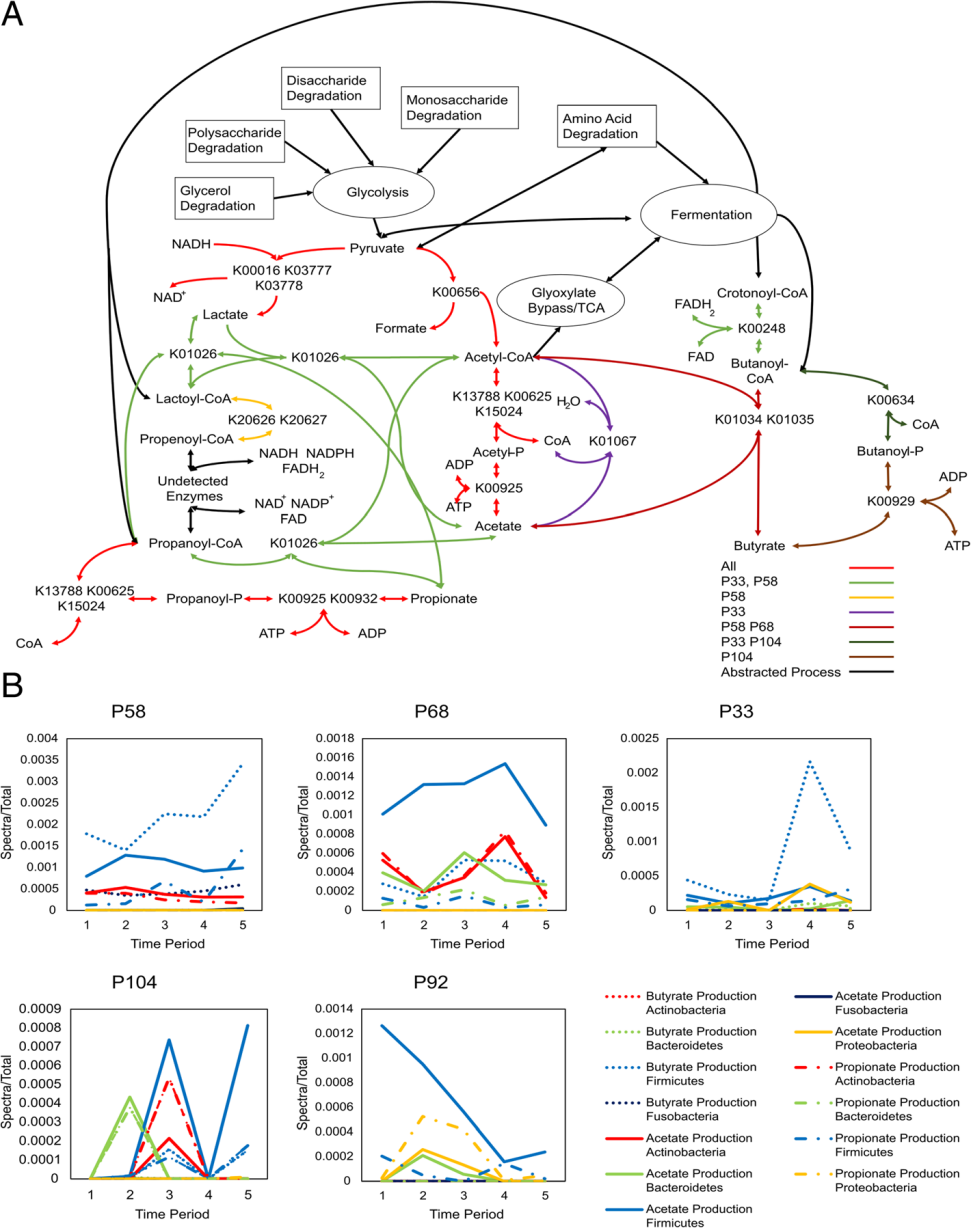
**a** Representation of direct routes to short-chain fatty acids and acetyl-CoA with proteomic evidence based on GOMixer modules. All KEGG Ortholog terms mentioned in this figure had proteomic evidence in at least one sample, although many had evidence across all individuals and most samples. For example, K00656 had proteomic evidence in all samples and in Firmicutes, Actinobacteria, Proteobacteria, and Bacteroidetes, but not always at the same time. These enzymes form a sizable representation of the pathways to these metabolites with proteomic evidence; however, not all enzymes that can produce these molecules are included. **b** Line graphs that depict the abundance of modules related to each short-chain fatty acid across time per individuals. The abundance of the modules related to each short-chain fatty acid were summed by phylum and divided by total number of spectra

An inspection of the inferred modules reveals several interesting points about the gut microbiome’s metabolic function in these five individuals. First, despite substantial variation in the protein groups observed in each sample, these modules demonstrate persistence of metabolic function across time and individuals. Sixty-nine modules were observed in all individuals, of which 18 were observed in all samples. Tracking through these 18 modules reveals a clear path from carbohydrate, lipid, and amino acid degradation to central metabolism and finally the production of fermentation products (Fig. 3). Second, this persistence of function is redundant across multiple phyla but was not always observable in the same sample. All of the 69 modules except for one were observed in two or more phyla, and all of the 18 modules observed across all samples were observed in two or more phyla, but not always in the same sample. In some cases, the protein evidence for a module comes from one phylum but then changes to another phylum at a different time. (Fig. 4b, Fig. 5b, Additional file 4: Spreadsheet 3, Additional file 1: Figures. S5-S9). Third, the gut microbiome metabolism is not driven by a set of discrete linear pathways but a web of interconnected reactions facilitated by a network of enzymes that connect multiple molecules across multiple pathways. For example, the identified protein groups suggest that lactose (MF0048) degrades into glucose before following the classical glycolysis pathway, while glycerol (MF0107, MF0108, MF0109) and fucose (MF0124) degrade into glyceraldehyde 3-phosphate, which is the precursor to the pay-off phase of glycolysis. Similarly, protein enzyme pathway detection suggests that threonine degradation (MF0029 and MF0030) skips glycolysis by degrading directly into acetyl-CoA and other fermentation products including propionate and formate.

These modules represent a major portion of the metabolic function of the gut microbiome. Population of these modules from the measured proteomes provide evidence for microbial-driven degradation of complex molecules across multiple samples, including all 20 amino acids, and a large repertoire of carbohydrates, such as starch, fucose, xylose, arabinose, lactose, and glycerol. Degradation modules connect to energy producing modules such as glycolysis, glyoxylate bypass, and fermentation, for which there was consistent protein evidence. These energy-producing modules culminate into a variety of gut relevant metabolites, including pyruvate, acetyl-CoA, succinate, formate, propanoyl-CoA, CO_2_, acetate, butyrate, and propionate.

The metaproteomic experimental evidence for these specific metabolic modules reveals a detailed functional view of the gut microbiome ecosystem, in which microbes acquire energy by degrading complex molecules from both the host and the host’s diet, and then this degradation process ultimately leads to metabolites, such as butyrate, acetate, and propionate, that the host can use for energy. It appears that the majority of this metabolic function is not specific to an individual bacterial strain, species, genus, or phylum but is instead shared between phyla. Furthermore, the phylum driving an individual module, as evidence by proteomic data, can change dynamically across time and individuals (Figs. 4b**,** and 5b**,** Additional file 1: Figure S5-S9). This manuscript provides a detailed analysis of a few of these key modules, but the full set of modules that were inferred with proteomic evidence across time and individuals is detailed in Additional file 4: Spreadsheet 3.

### Observation of acetylglucosamine and acetylneuraminate indicate the microbiome can feed itself at the expense of the host

Detailed analysis of Fig. 3, and the information in Additional file 4: Spreadsheet 3, prompted interest in two specific modules, MF003 and MF005. These modules are observed in all individuals across multiple phyla and highlight metabolites that enter central metabolism at multiple positions in the pathway. Specifically, these modules represent the degradation of acetylglucosamine and acetylneuraminate into d-Fructose 6-phosphate, pyruvate, and acetate. Acetylglucosamine and acetylneuraminate are sugar amine hybrids found in the host mucosal layer, human milk oligosaccharides, and in animal food products that are key host nutrients and presumably are used by the gut microbiome as a source of energy [44–46]. Interestingly, providing N-acetyl-glucosamine as a supplement has been suggested as a potential method for alleviating inflammatory bowel disease [47]. MF0003 and MF0005 enter central metabolism at three separate levels, and merge with each other at N-acetyl-d-glucosamine 6-phosphate, highlighting a simple demonstration of the interconnected nature of gut microbiome metabolism (Fig. 4).

Evidence for the presence of MF003 and MF005 was observed across all individuals and in Firmicutes, Proteobacteria, Bacteroidetes, and Actinobacteria, thereby demonstrating the persistence of function despite the dynamic nature of the gut microbiome. Evidence for N-acetylneuraminate lyase (K01639) was observed in Firmicutes (P68, P92, P58, and P104), Proteobacteria (92), and Bacteroidetes (P68, P33, P58, and P104) but not in Actinobacteria. Evidence for the production of N-Acetyl-d-glucosamine 6-phosphate was limited in both modules; however, evidence for N-acetylmannosamine-6-phosphate 2-epimerase and N-acetylmannosamine kinase (K00885 and K01788) was observed in Proteobacteria on day 88 in P92 and in Actinobacteria on day 173 in P104. Similarly, evidence for the phosphotransferase system (K02802, K02803, and K02804) was only observed in Firmicutes in individuals P68 and P104, and in both Firmicutes and Proteobacteria in individual P92. Despite the relatively sparse evidence for enzymes specifically involved in the production of N-acetyl-d-glucosamine 6-phosphate, mentioned above, there was substantial evidence for enzymes associated with the utilization of N-acetyl-d-glucosamine 6-phosphate. N-acetylglucosamine-6-phosphate deacetylase (K01443) was observed across all individuals in Firmicutes (P68, P92, P33, P58, P104), Proteobacteria (P92), Actinobacteria (P33, P58, P104), and Bacteroidetes (P68, P92, P33, P104). Glucosamine-6-phosphate deaminase (K02564) was also observed across all individuals in Firmicutes (P68, P92, P33, P58, P104), Actinobacteria (P68, P58, P104), Proteobacteria (P92), and Bacteroidetes (P68, P92, P33, 58, P104). Together, these enzymes show consistent presence of both modules across all individuals.

Of interest, N-acetylglucosamine-6-phosphate deacetylase (K01443) was highlighted as a core enzyme in a previous healthy cohort study [24]. Combined with the fact that this enzyme is found across multiple phyla, this suggests that it may be a key source of energy for gut microbiomes. In contrast, K01639 and K01639 were also observed in all individuals in this study but were not in the core set from this previous study, possibly suggesting a potentially different utilization of d-glucosamine 6-phosphate.

These enzymes serve as a representative case for both the redundant and dynamic nature of microbiome metabolic functions. For example, most of these enzymes were observed in one of the four phyla at least once, but the modules were not always inferred in the same phyla across individuals. These modules also highlight the web like nature of gut microbiome metabolism. The degradation of acetylglucosamine and acetylneuraminate connects at N-acetyl-d-glucosamine 6-phosphate, and together these modules release products into central metabolism at three separate points: d-fructose 6-phosphate, pyruvate, and acetate. d-fructose 6-phosphate is a precursor to the pay-off phase of glycolysis. Pyruvate is the end product of the pay-off phase of glycolysis and is the starting point for a variety of fermentation pathways, as well as the TCA cycle and glyoxylate bypass. Acetate is one of the three main short-chain fatty acids produced by the gut microbiome and is a source of energy for both the host and the microbiome. It can be converted to acetyl-CoA at the cost of ATP or via transferase with the help of butanoyl-CoA or propanoyl-CoA [48]. Conversion by transferase produces butyrate and propionate which can also be converted by the host into energy. Together, these modules represent a sample case of how many different phyla contain similar metabolic modules that can utilize host derived molecules, such as acetylglucosamine and acetylneuraminate.

### Observation of short-chain fatty acid associated modules indicates that the host is fed by a changing set of pathways and bacteria

Just as acetylglucosamine and acetylneuraminate feed the microbiome, short-chain fatty acids, particularly acetate, propionate, and butyrate, are thought to be one of the primary methods by which the gut microbiome feeds its host. “GOmixer” highlights nine modules as acetate (MF0113), propionate (MF0121, MF0122, MF0123, MF0125, MF0126), or butyrate (MF0114, MF0116, MF0117) producing. All these modules were observed with peptide evidence in at least one sample. Some of these modules do not represent the direct production of short-chain fatty acids but instead represent the production of precursors to CoA-transferase and kinase reactions. Only MF0113, MF0116, MF0117, MF0125, and MF0126 connect the direct production of short-chain fatty acids via CoA-transferase, kinase, or a similar reaction. The average abundance of the KO terms in each module divided by the total depicts the persistence of function amidst a dynamic microbial ecosystem as evidence for particularly propionate and acetate producing modules persistence across time and individuals, while being driven by different phyla or modules (Fig. 5b, Additional file 1: Figures. S5-S7; Additional file 4: Spreadsheet 3). Evidence for enzymes involved in the direct production of butyrate was observed only in individuals P58, P68, and P92. This supports previous proteomic studies, which indicated that the abundance of butyrate producing enzymes is significantly lower in patients with Crohn’s disease [18].

A reaction network was manually produced by utilizing the KEGG orthologous groups that had representation in the modules and their associated reactions, as seen in Fig. 5a. This network highlights evidence for a dynamic and interconnected microbial metabolic function. Acetate production via acetate kinase (MF0113) had peptide evidence across all individuals. One set of enzymes can produce acetate via a two-step process that involves first converting acetyl-CoA to acetyl-phosphate (K13788, K00625, K15024) and then converting acetyl-phosphate to acetate (K00925), producing ATP in the process (Fig. 5a). These reactions are reversible [49], and there was evidence for the enzymes involved in both steps across all individuals and almost all the samples. According to the KEGG database and the literature, given propanoyl-CoA as the starting material, this module will also produce propionate, as observed from peptide evidence for the potential production of propionate across all individuals [50]. This highlights the interconnectedness of gut metabolism, as the same enzymes can plug into different fermentation routes of the broader metabolic network given different starting material.

There was also limited evidence for a propionate unique kinase (K00932) in individual P92 and the production of propionate via CoA transferase (K01026) in P33 and P58. Production of butyrate or propionate by CoA-transferase requires the transfer of the coenzyme from butyrate or propionate to another acid typically acetate, but it could also be lactate. This is an interconnected process. For example, propionate CoA-transferase, K01026, according to KEGG reactions, transfers CoA between lactate, propionate, or acetate to produce lactoyl-CoA, acetyl-CoA, or propanoyl-CoA (Fig. 5). K01026 further highlights the interconnectedness of gut microbiome metabolism, as Proponyl-CoA is converted into propionate by converting acetate or lactate into acetyl-CoA or lactoyl-CoA respectively, and vice versa.

Butanoyl-CoA can be converted into butyrate by two main methods, presenting a good example of the dynamics of microbial metabolic function. Acetate CoA-transferase (K01034 and K01035) converts Butanoyl-CoA to butyrate by converting acetate into acetyl-CoA and vice versa. Butyrate kinase is a two-step process that involves conversion of butanoyl-CoA to butanoyl-phosphate (K00634), followed by the production of butyrate via the kinase (K0929). In each individual, except P92 which had no peptide evidence of butyrate production, only one method or the other was observed. P58 and P68 had evidence for the production of butyrate via transferase, and P104 and P33 had evidence for the production of butyrate via kinase. P33 only had peptide evidence for K00634 but not the actual kinase. This presents an example of persistence of function despite a dynamic gut microbiome. There was peptide evidence for butyrate production across most individuals.

Although not typically included in discussions about short-chain fatty acid production in the gut, it should be noted that peptide evidence for the production of the smallest short-chain fatty acid, formate, was observed via K00656 across all samples, thus providing persistent peptide evidence for the conversion of pyruvate to acetyl-CoA across a variety of phyla.

K00656 has been consistently observed in previous metaproteomic studies, along with acetate producing enzymes K00625 and K00925, [24, 43] indicating that acetate production via these enzymes may be ubiquitous regardless of human host health status. Interestingly, one of these studies [24] highlights propionate CoA-transferase and butryl-CoA dehydrogenase as being core enzymes, although these are not observed across all individuals in this dataset. This correlates to previous research which has shown that short-chain fatty acid-producing proteins, especially butyrate, are less abundant in Crohn’s disease patients [18].

## Conclusions

Measurement of the metaproteomes of Crohn’s disease patients (all post resection-surgery and in remission) over the course of a year revealed substantial variability in the protein groups observed across time and individuals. There was significant qualitative variability even within the same individuals, which supports the dynamic nature of the microbiome’s composition that was previously observed in the same individuals [26]. The gut metaproteomes of these individuals were distinctly personalized, with some individuals exhibiting less variability across time than others.

This study demonstrated that despite this variability, many metabolic functions are consistently observed across diverging sequence space, suggesting that sequential variability may not be a good indicator of metabolic functional variability. Individual KO terms were found to be more consistent across time and individuals than protein groups. The majority of KO terms observed originated from Firmicutes, Actinobacteria, Proteobacteria, and Bacteroidetes, and when placed in a metabolic context, consistent and somewhat redundant metabolic functions relating to the degradation and fermentation of food products were observed.

Although this study did not focus on taxonomical resolution below the phylum level, it is evident that almost all the functions observed across all individuals were observed in multiple phyla. This provides compelling evidence that these functions are not specific to any one phylum, genus, or species and may suggest that functional redundancy across taxa is a hallmark of robust gut microbiome stability. These modules reveal the interconnectedness of gut microbiome metabolism, suggesting that overall gut microbiome operation should be viewed in a network context focused on metabolic function.

Clearly, the integration of metagenomic assembled genomes (MAGs) with deep metaproteome data would provide specific details down to the strain level and thus enable high-resolution metabolic pathway reconstruction at the most informative level. While this is a desirable goal, in reality, the construction of extensive MAGs in complex microbiomes is still in its infancy. In most cases, it is beyond current state-of-the-art to accomplish this goal. Thus, one is left with a range of complete and incomplete taxa assignments, all of which confound the integrated metagenomic/metaproteome datasets by introducing large uncertainties and variabilities in assigned genes/proteins to taxa. Inspection of our metaproteome datasets at various taxonomic levels revealed that the most informative data about functional activity was at the phyla level, where metabolic activity was found to be driven by temporal dynamic and redundant phyla representation. Although this is a broad level view, even this analysis provides novel insights into robust and temporally redundant microbiome operation.

Although this study was not designed to directly contrast healthy individuals versus individuals with Crohn’s disease, these specific diseased individuals have been contrasted with healthy individuals in a prior study [26] and thus were selected here to encompass the widest range of taxonomical variability. The data revealed that microbiomes of these post-surgery individuals had significant variability in taxa, genes, and proteins; however, key metabolic modules associated with central metabolism were seen in all samples, even though the phyla of origin was often different. End-point metabolic modules, such as short-chain fatty acid production, were seen intermittently across samples, although only butyrate-producing modules were absent in one individual.

Specifically, there was evidence that there is persistent and phylum-redundant metabolic functional stability in these individuals. This approach provides a distinct observational viewpoint of following metabolic reactions and compounds/enzymes as a marker of overall microbiome activity, even if populations and specific genes/proteins vary.

## Additional files

Additional file 1: Appendix 1 and Supplemental figures. (DOCX 7204 kb)

Additional file 2: SpreadSheet1_ProteinGroupingAndAnnotation.tab. (TXT 1429 kb)

Additional file 3: SpreadSheet2_IdentifiedProteinGroupsSpectralCountsPerRun.txt. (TXT 2805 kb)

Additional file 4: SpreadSheet3_GOMixerInferredModules0.333CoveragePerPhylum.txt. (TXT 73 kb)

## Abbreviations

DTT: Dithiothreitol
FDR: False discovery rate
KEGG: Kyoto Encyclopedia of Genes and Genomes
KO: KEGG orthology group
SCFA: Short-chain fatty acid
SDS: Sodium dodecyl sulfate
TCA: Trichloroacetic acid
