# Supplementary material for: Metaproteomics reveals persistent and phylum-redundant metabolic functional stability in adult human gut microbiomes of Crohn’s remission patients despite temporal variations in microbial taxa, genomes, and proteomes
Source: Microbiome. 2019 Feb 11;7:18. doi: 10.1186/s40168-019-0631-8 (PMC6371617; doi:10.1186/s40168-019-0631-8)
Supplement: Supplementary file 1 — Appendix 1 and Supplemental figures. (DOCX 7204 kb) [file 40168_2019_631_MOESM1_ESM.docx]

**Metaproteomics reveals persistent and phylum-redundant metabolic functional stability in adult human gut microbiomes despite temporal variations in microbial taxa, genomes, and proteomes**

J. Alfredo Blakeley-Ruiz^1,2^, Alison R Erickson^1^, Brandi L Cantarel^3^, Weili Xiong^1^, Rachel Adams^1^, Janet K Jansson^5^, Claire M Fraser^3, 4^, and Robert L Hettich^1,2,*^

**^1^** Chemical Sciences Division, Oak Ridge National Laboratory, Oak Ridge, TN 37831, USA.

**^2^** Graduate School of Genome Science & Technology, University of Tennessee, Knoxville, TN 37996, USA.

**^3^** Institute for Genome Sciences, University of Maryland School of Medicine, Baltimore, MD 21201, USA.

^4^Department of Medicine, University of Maryland School of Medicine, Baltimore, MD 21201, USA.

**^5^** Biological Sciences Division, Pacific Northwest National Lab, Richland, WA, 99352.

**Appendix 1: A Description of the Seven Microbial Proteins Identified in All Samples**

While variation was observed within individuals at the protein group level, it was even higher between individuals. Out of 14,850 protein groups, only 60 were identified in all samples. Of those protein groups, 53 were human, and the remaining 7 were microbial. All of these microbial protein groups mapped to the genus *Bacteroides*, and, in most cases, these protein groups were relatively low abundance in the sample, often having less than 100 spectral counts (**Supplementary Figure 10**).

All 7 of these protein groups were queried for their known function and conserved domains using UniProt and InterPro Scan respectively (Supplementary Figures 12-19) [1-3]. Three of these protein groups (104293, 93252, 16954) were found to be related to metabolism. ProteinGroup_104293 had the most spectral counts and contained two domains related to Glyceraldehyde 3-phosphate dehydrogenase, which catalyzes one of the reducing steps in glycolysis (**Supplementary Figure 11**). ProteinGroup_16954 contains a FAD binding domain and is annotated as a succinate dehydrogenase/fumarate reductase subunit, which reduces fumarate into succinate or oxidizes succinate into fumarate, and according to KEGG is involved in a variety of microbial metabolic processes, including the TCA cycle, short chain fatty acid metabolism, carbon fixation, and carbon metabolism (**Supplementary Figure 12**) [4]. ProteinGroup_93252 is annotated as phosphoserine aminotransferase, which according to KEGG is a component of the transition from Glycerate 3-phospate to serine and vice versa, and as a result plays a role in the degradation and biosynthesis of amino acids (**Supplementary Figure 13**). Together these three protein groups cover a wide range of microbial metabolism including central metabolism, fermentation, and amino acid metabolism.

Of the remaining 4 protein groups, ProteinGroup_253057 is annotated as a L5 ribosomal protein (**Supplementary Figure 14**), while protein groups 28742, 171203, and 3177 are proteins of limited known function with low quality annotations based on their domains. ProteinGroup_28742 could potentially be an antigen. All the sequences that make up this protein group contain a tetratricopeptide repeat domain (**Supplementary Figure 15**). This domain is a common domain often involved in protein-protein interactions and is an important component of several virulence factors among other types of proteins [5]. Along those lines, the submitted name for one of the perfect hits in UniPropt for the sequences clustered under ProteinGroup_28742 was “Immunoreactive 53 kDa antigen PG123”. Further research is necessary to fully characterize the function of these sequences. The sequences clustered under ProteinGroup_3177 contained TonB dependent receptor domains and are likely to be a part of the SusC/RagA family (**Supplementary Figure 16**). Interestingly, TonB and SusC were shown to be significantly more abundant in ileal Crohn’s disease in a previous publication [6]. The UniPropt hits to the sequences clustered under ProteinGroup_171203 were typically either “Uncharacterized protein” or “NifU-related domain containing protein”. Linking from the UniPropt annotation to the InterPro annotation revealed that the sequences clustered under this protein group were homologous to the domain labeled IPR002871 (**Supplementary Figure 17**). This domain is reported to be the N-Terminal of NifU, which contains a 2Fe-2S cluster, and is implicated to be involved in nitrogen fixation systems, although the exact biochemical function remains unknown[3, 7]. A homolog to this domain was highlighted previously as a core human gut metaproteome protein [8]. Given its robust observation in gut metaproteome studies, further insight into the function of this family of proteins could reveal novel insight into gut community function. The realization that almost half of the proteins identified across all samples in our database remain poorly characterized highlights the challenge of using proteomics to understand microbiome function when many if not most of the protein sequences remain poorly understood.

**Supplementary Figures:**

**Supplementary Table 1**. Sample Description. The MG column indicates whether the metagenome was sequenced. AB indicates whether antibiotics were used, and if antibiotics were used in the 30 days prior to sampling.

| Patient # | Sample Name | Day | AB | MG | Birth Year | Gender | Smoker | Extent of Disease | Stricturing |
| --- | --- | --- | --- | --- | --- | --- | --- | --- | --- |
| 33 | A022 | 0 |  | Yes | 1944 | F | Yes | Ileocolon | No |
| 33 | A64 | 82 |  |  |  |  |  |  |  |
| 33 | A120 | 175 |  | Yes |  |  |  |  |  |
| 33 | A177 | 259 |  |  |  |  |  |  |  |
| 33 | B75 | 361 |  | Yes |  |  |  |  |  |
| 33 | B190 | 441 | Y |  |  |  |  |  |  |
| 58 | A49 | 0 |  | Yes | 1949 | F | No | Ileocolon | Yes |
| 58 | B93 | 73 |  |  |  |  |  |  |  |
| 58 | A150 | 171 |  | Yes |  |  |  |  |  |
| 58 | B11 | 263 |  |  |  |  |  |  |  |
| 58 | B135 | 352 |  | Yes |  |  |  |  |  |
| 68 | A52 | 0 | Y | Yes | 1952 | M | Ex | Ileocolon | Yes |
| 68 | A105 | 91 | Y |  |  |  |  |  |  |
| 68 | A158 | 175 | Y | Yes |  |  |  |  |  |
| 68 | B40 | 274 | Y |  |  |  |  |  |  |
| 68 | B152 | 357 |  | Yes |  |  |  |  |  |
| 92 | A42 | 0 |  | Yes | 1961 | F | Yes | Terminal Ileum | Yes |
| 92 | A91 | 88 |  |  |  |  |  |  |  |
| 92 | A144 | 177 |  | Yes |  |  |  |  |  |
| 92 | B6 | 282 |  |  |  |  |  |  |  |
| 92 | B125 | 367 |  | Yes |  |  |  |  |  |
| 104 | A30 | 0 |  | Yes | 1967 | M | No | Terminal Ileum | Yes |
| 104 | A77 | 84 |  |  |  |  |  |  |  |
| 104 | A131 | 173 |  | Yes |  |  |  |  |  |
| 104 | A194 | 259 | Y |  |  |  |  |  |  |
| 104 | B99 | 362 | Y | Yes |  |  |  |  |  |
| 104 | B187 | 427 |  |  |  |  |  |  |  |

**Supplementary Figure 1**: Box plots of the (A) the number of microbial (blue), human (orange), and total (grey) protein groups per sample and (B) the number of spectral counts assigned to each of those groups per sample.


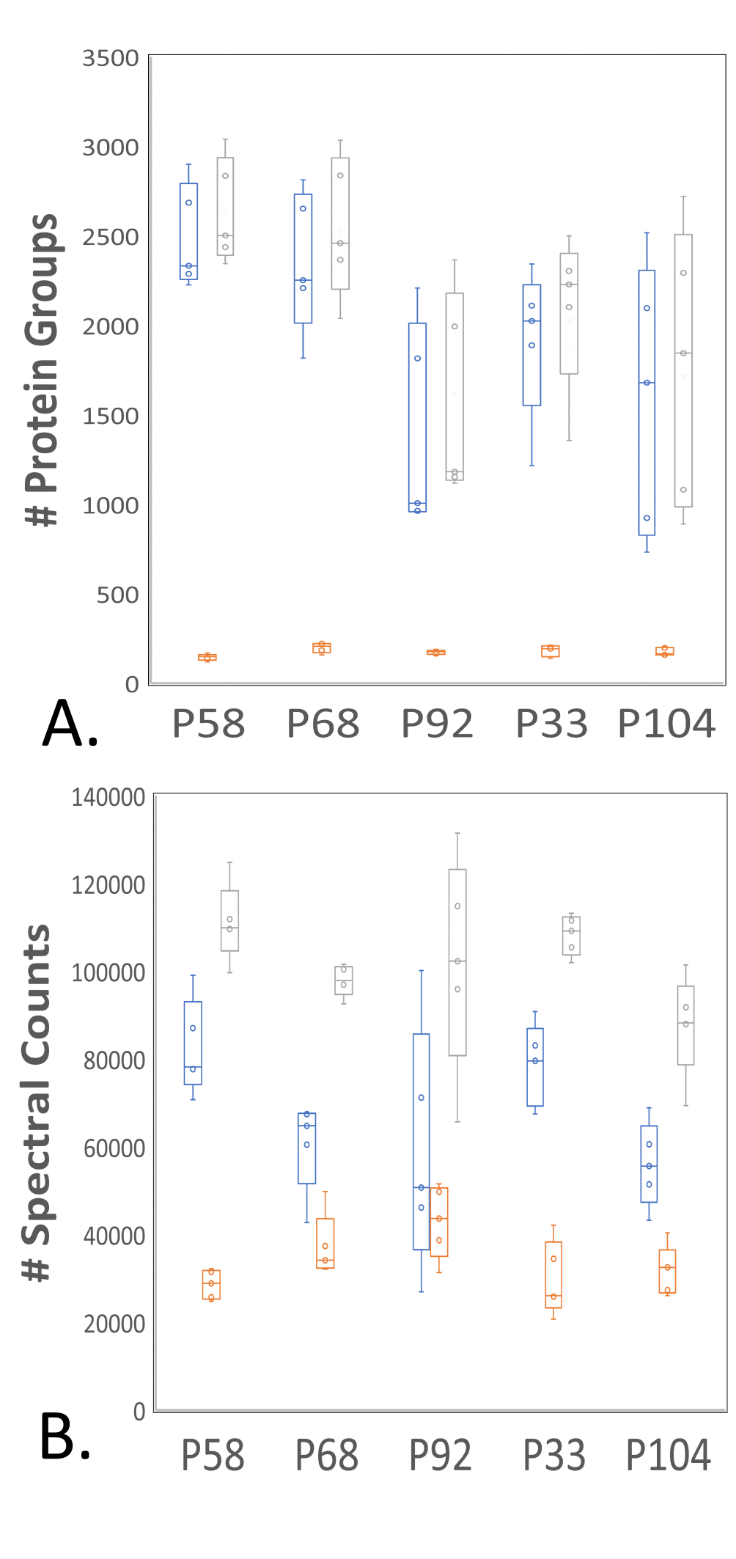


**Supplementary Figure 2**: Technical reproducibility from several perspectives. (A) Distribution of Spearman Correlation Index of raw spectra, Pearson Correlation Index of Log2 transformed spectra, and Pearson Correlation Index of raw spectra for each sample. (B) Percentage of the total protein groups identified in each sample that were found in both technical duplicates. (C) Linear fit of raw spectra identified in both technical replicates of sample P92_d177. Sample P92_d177 had the highest technical reproducibility per Spearman correlation index. (D) Linear fit of raw spectra identified in both technical replicates of P104_d259. P104_d259 had the lowest spearman correlation, while P92_d177 had the highest spearman correlation between technical replicates.
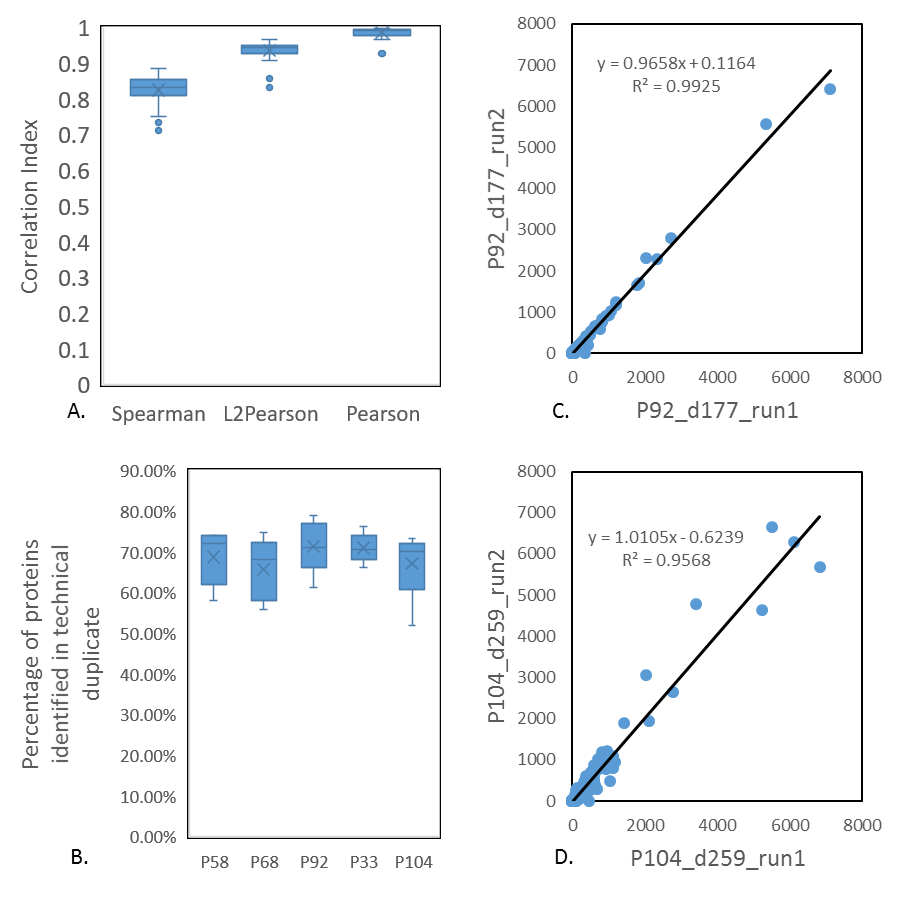


**Supplementary Figure 3**: Comparison between the metagenome derived protein database and the metaproteome for each individual and sample. (A) Total number of open reading frames per individual specific protein database assigned to each Phylum via Ghost KOALA. (B) Total number of protein groups assigned to each phylum in each sample. In both A and B, light blue represents Firmicutes, orange represents Actinobacteria, grey represents Bacteroidetes, yellow represents Proteobacteria, and dark blue represents other phyla. (C) Represents the total number of KEGG Orthologous groups found in each sample individual protein database, in black, and the total number of KEGG Orthologous groups identified in the combined metaproteomes of each individual, in grey.


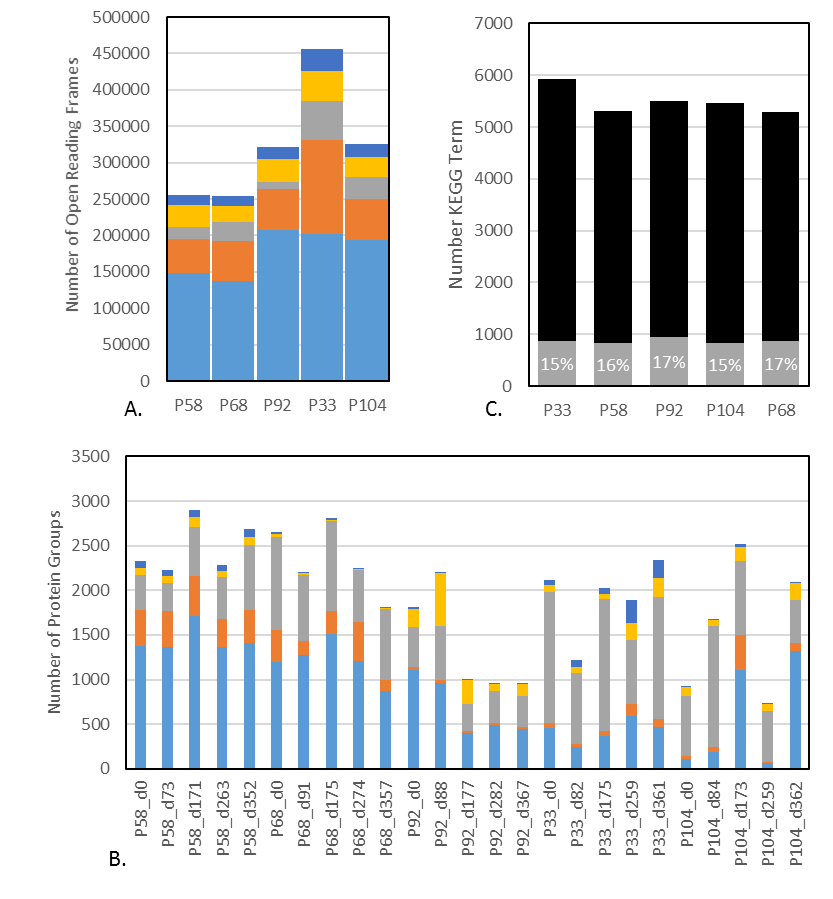


**Supplementary Figure 4**: Heatmap illustrating the ratio of total predicted KEGG orthologous groups that was identified by each phylum with peptide evidence in the metaproteomic data set. The ratio of total predicted KEGG orthologous groups was calculated by taking the total number of KEGG orthologous groups identified with peptide evidence in each phylum and dividing that by the total number KEGG orthologous groups predicted by that individual’s protein database. The first column indicates mean representation across all samples. The heatmap was rendered using the program Orange [9].


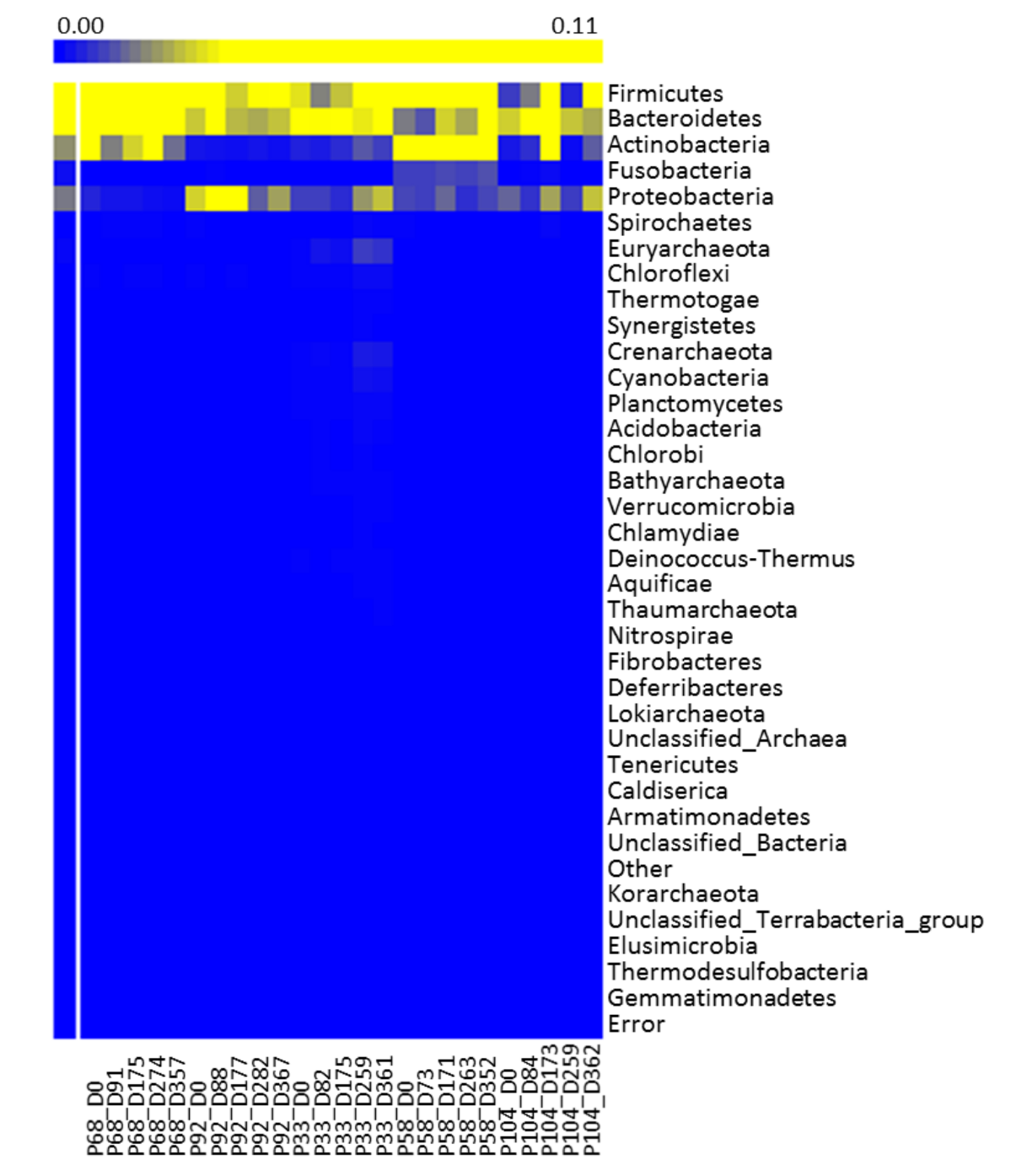


**Supplementary Figure 5**: There is only one GOMixer module that describes acetate production, MF0113. Here we represent trending across time of MF0113 in each individual by phylum.


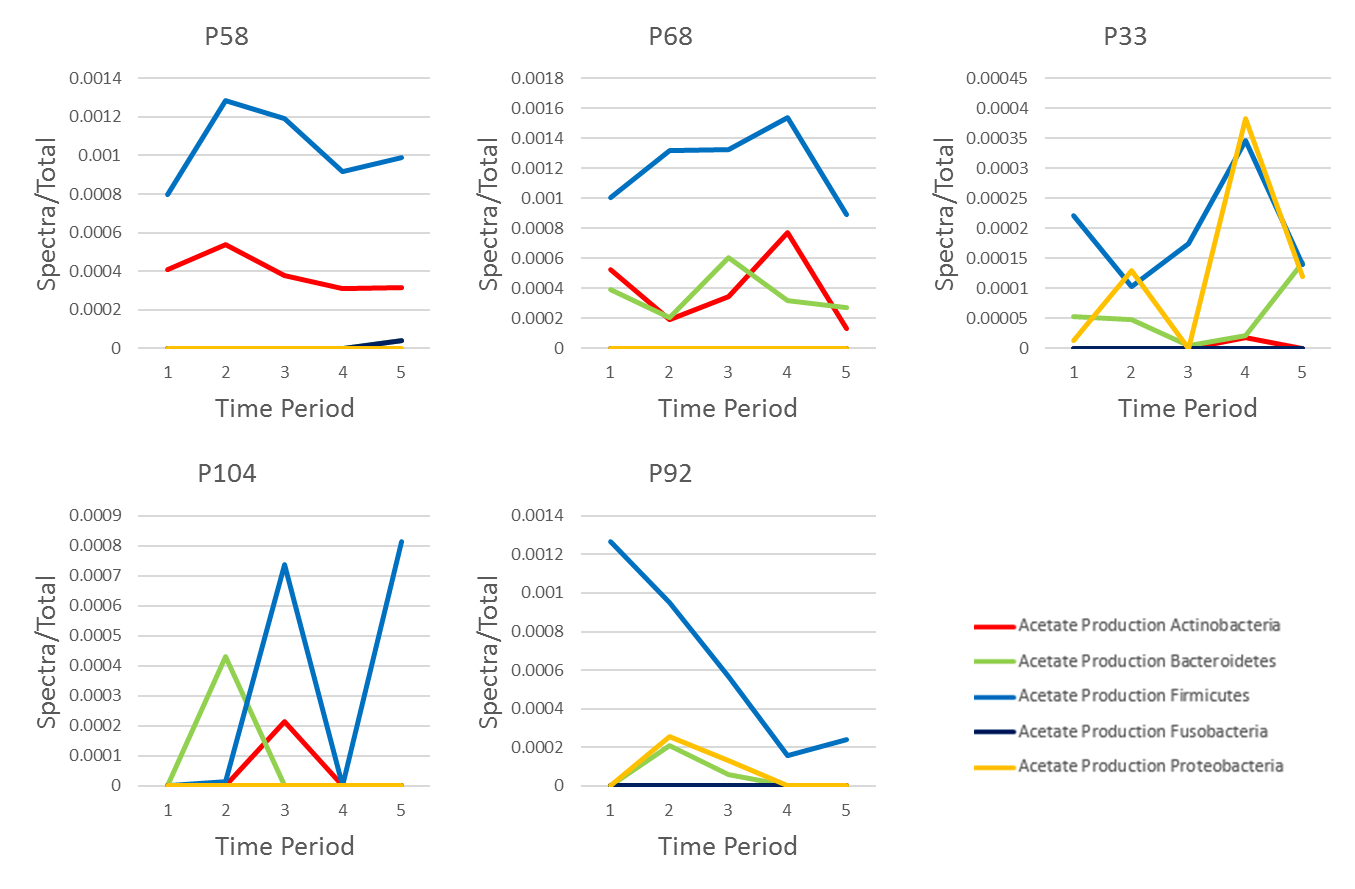


**Supplementary Figure 6**: Specific trends in butyrate production, separated by phylum, in three individuals where this was observed. There are two main modules inferred in our dataset that depict butyrate production MF0116 and MF0117 which represented butyrate production via transferase respectively and butyrate production via kinase respectively.


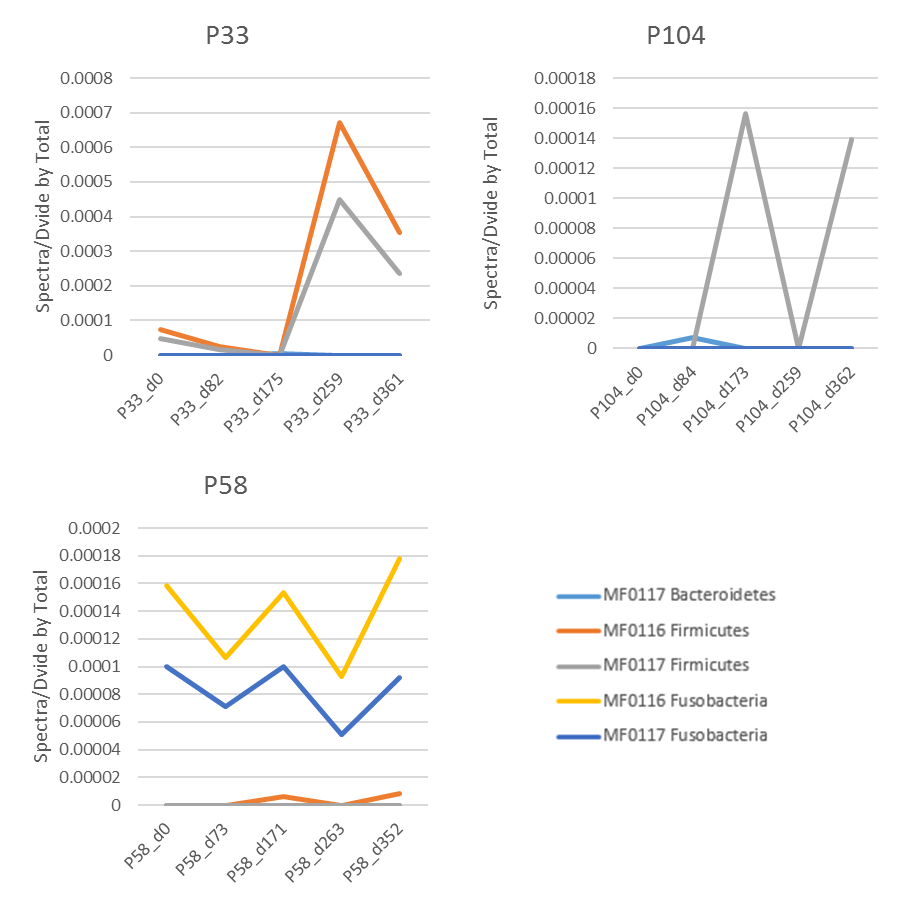


**Supplementary Figure 7**: Spectra divided by total by phylum of inferred modules depicting propionate production. Dashed lines represent modules that that are precursors to actual propionate production while the solid lines represent modules that produce propionate.


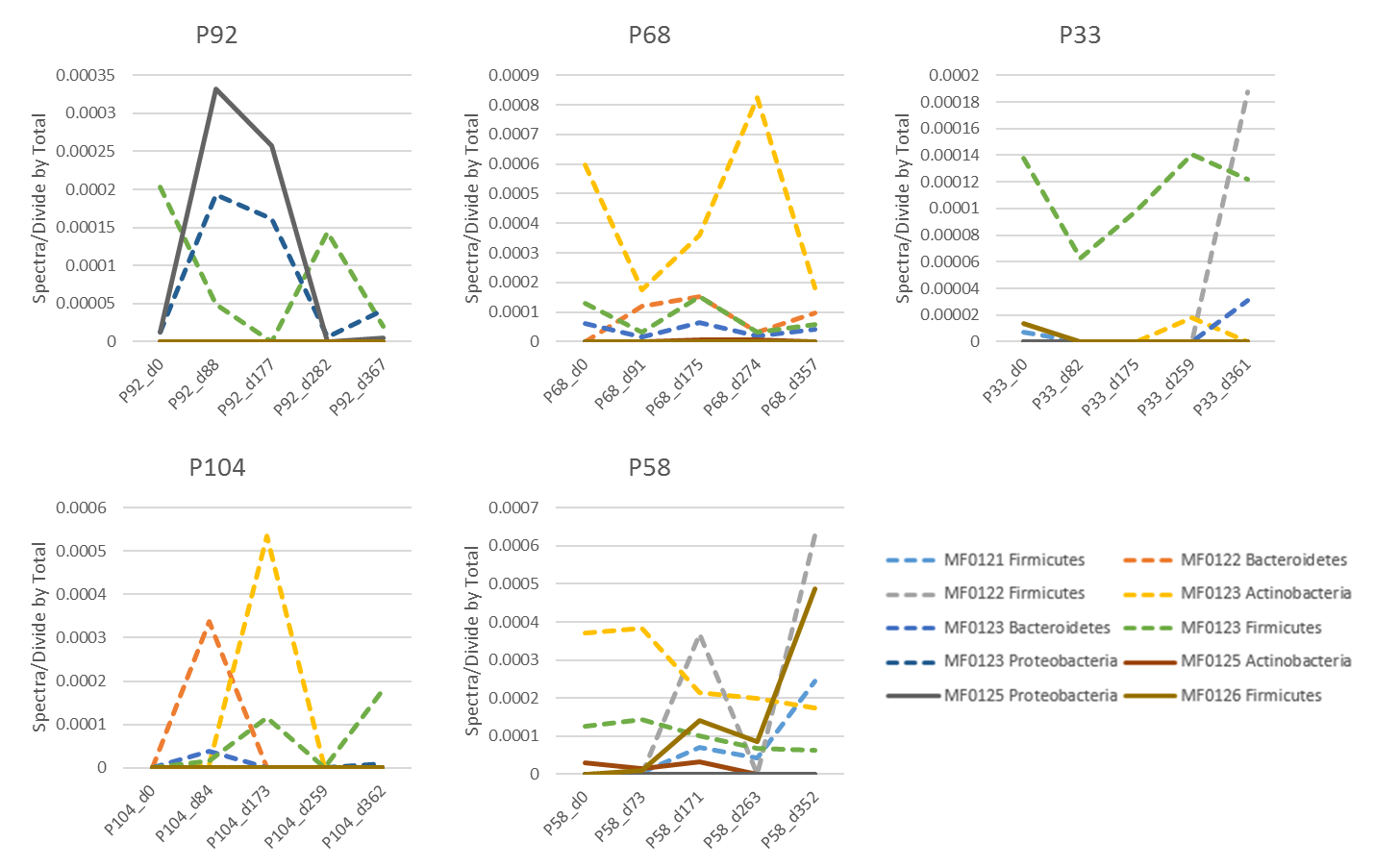


**Supplementary Figure 8**: Inferred modules for glycolysis by phylum. Dashed line represents the pay-off phase, MF0081, and the solid line represents the preparatory phase, MF0080.


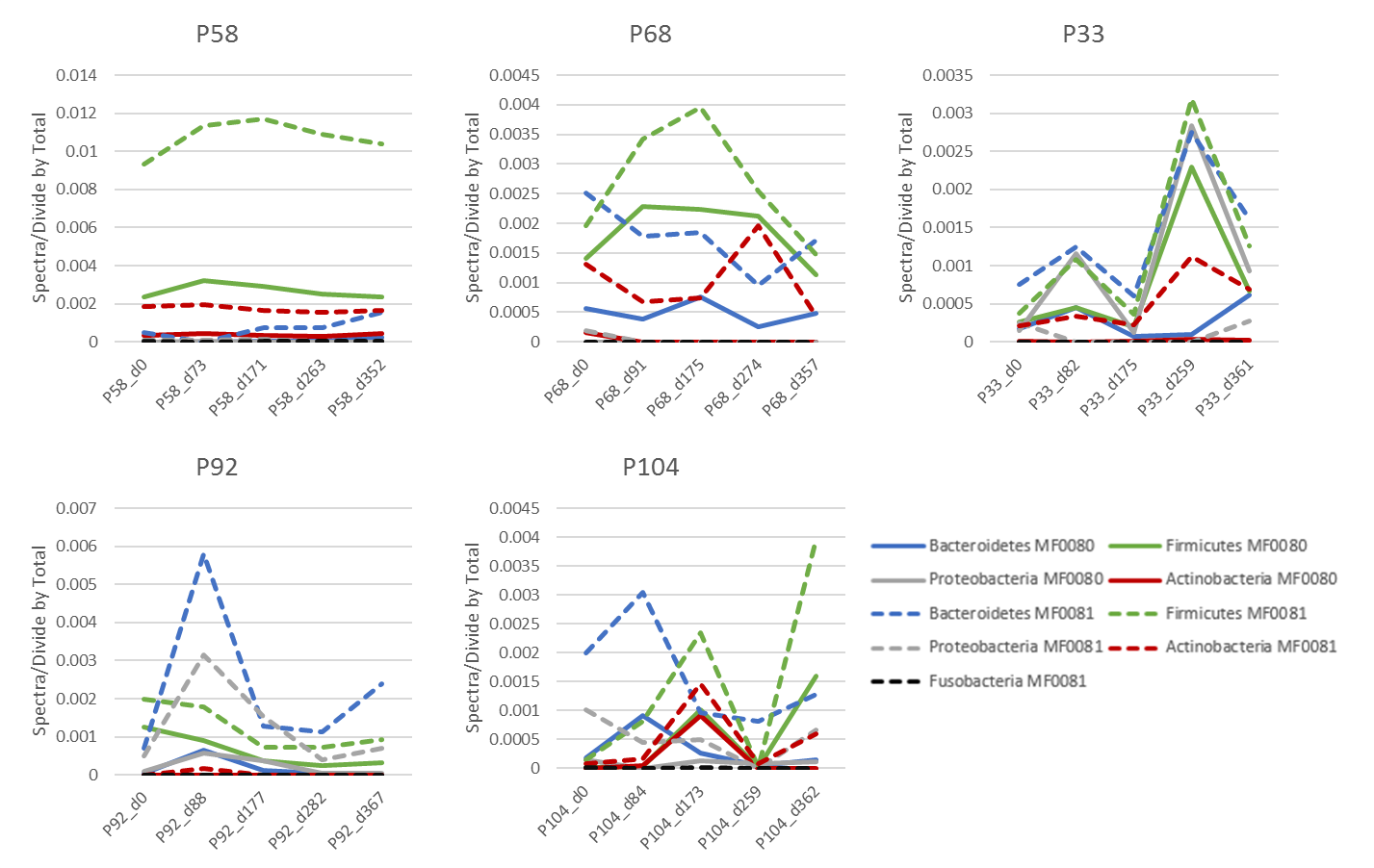


**Supplementary Figure 9**: Trends by phylum in modules pertaining to degradation of food products that were consistently observed across all samples. MF0029 and MF0030 represent different pathways that degrade Threonine the only amino acid degradation pathway that was seen across all samples. MF0048 represents Lactose degradation. MF0062 represents starch degradation a critical polysaccharide food source that comes from plants. MF0108 represents glycerol degradation, the only lipid degradation pathway that is observed across all samples. MF0124 represent Fucose degradation which is a mucosal glycan that the host produces and that the bacteria in the gut microbiome consume.


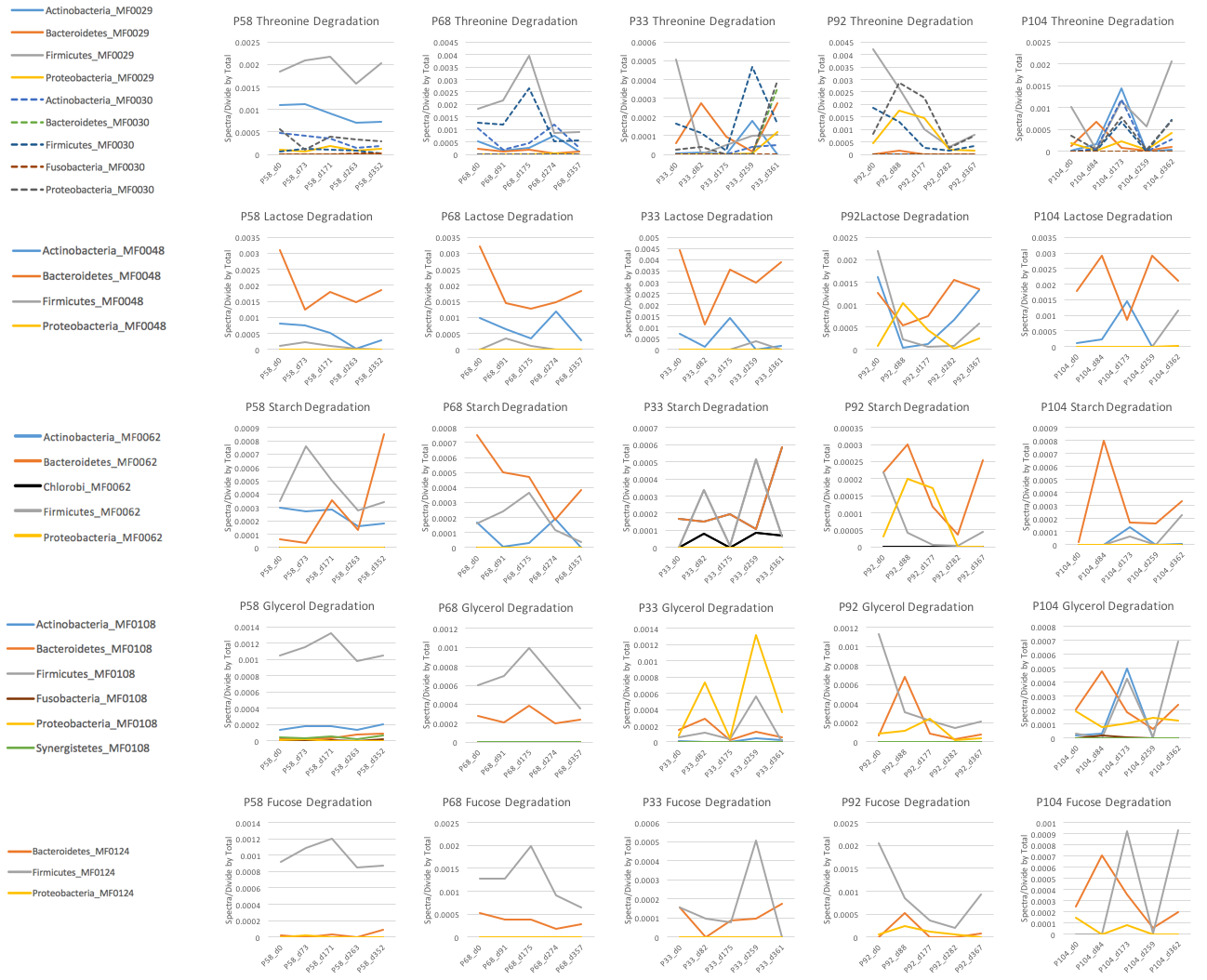


**Supplementary Figure 10**: Seven protein groups that were consistently observed across all samples


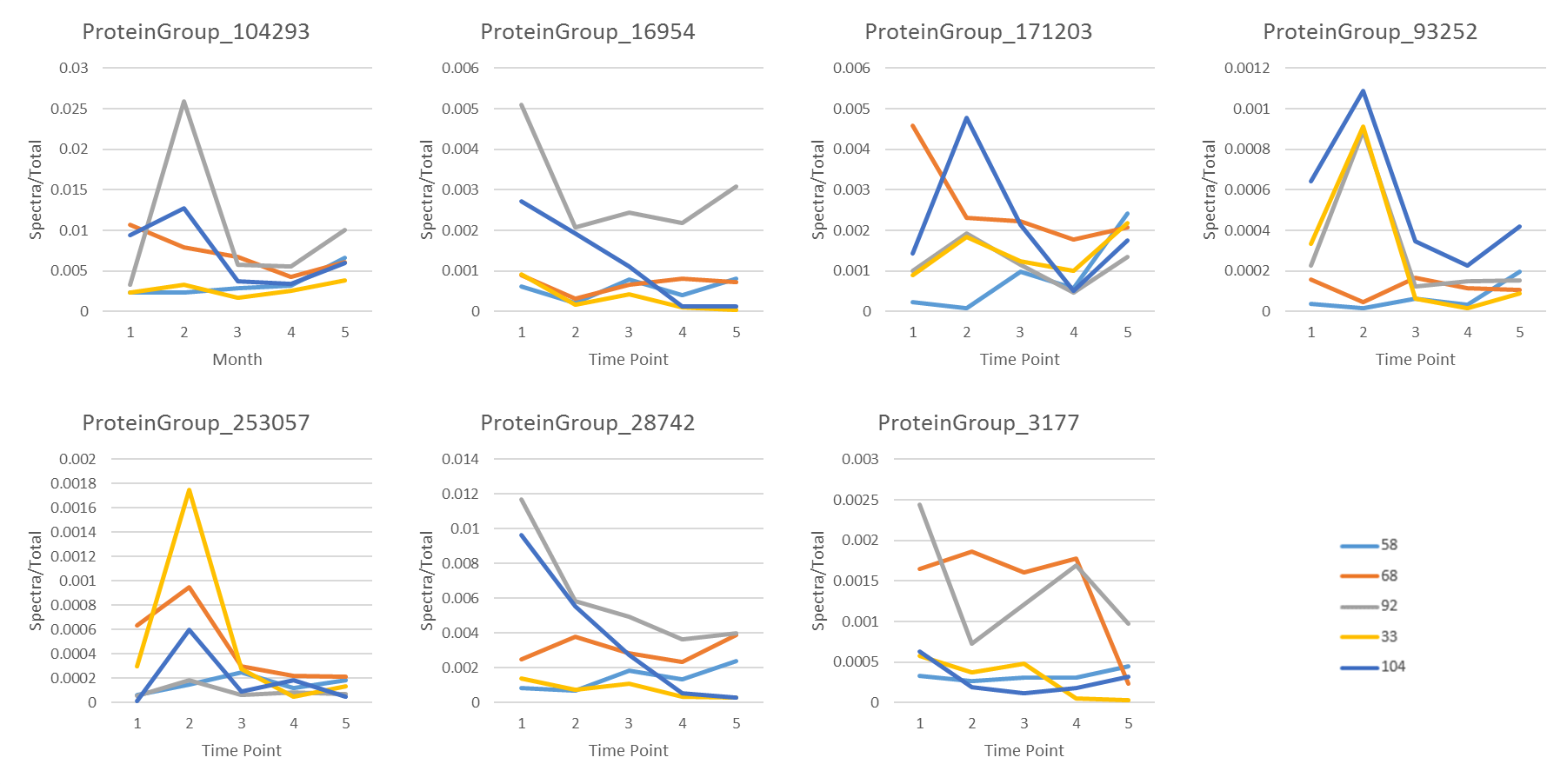


**Supplementary Figure 11**: InterPro Scan Result for ProteinGroup_104293 representative sequence. Sequence submitted 2/27/2018. Revealed domains IPR020828 and IPR020829 which are the NAD(P) binding and catalytic domains respectively for Glyceraldehyde 3-phosphate dehydrogenase.

>P58|orf11657 COG0057 Glyceraldehyde-3-phosphate dehydrogenase/erythrose-4-phosphate dehydrogenase

MIKVGINGFGRIGRFVFRAAMKRNDIQIVGINDLCPVDYLAYMLKYDTMHGQFDGTIEAD

VENSKLIVNGQAIRITAERNPADLKWNEVEAEYVVESTGLFLSKDKAQAHIEAGAKYVVM

SAPSKDDTPMFVCGVNEKTYVKGTQFVSNASCTTNCLAPIAKVLNDKWGITDGLMTTVHS

TTATQKTVDGPSMKDWRGGRAASGNIIPSSTGAAKAVGKVIPALNGKLTGMSMRVPTLDV

SVVDLTVNLAKPATYAEICAAMKEASEGELKGVLGYTEDAVVSSDFLGDTRTSIFDAKAG

IALTDTFVKVVSWYDNEIGYSNKVLDLIAHMASVNC


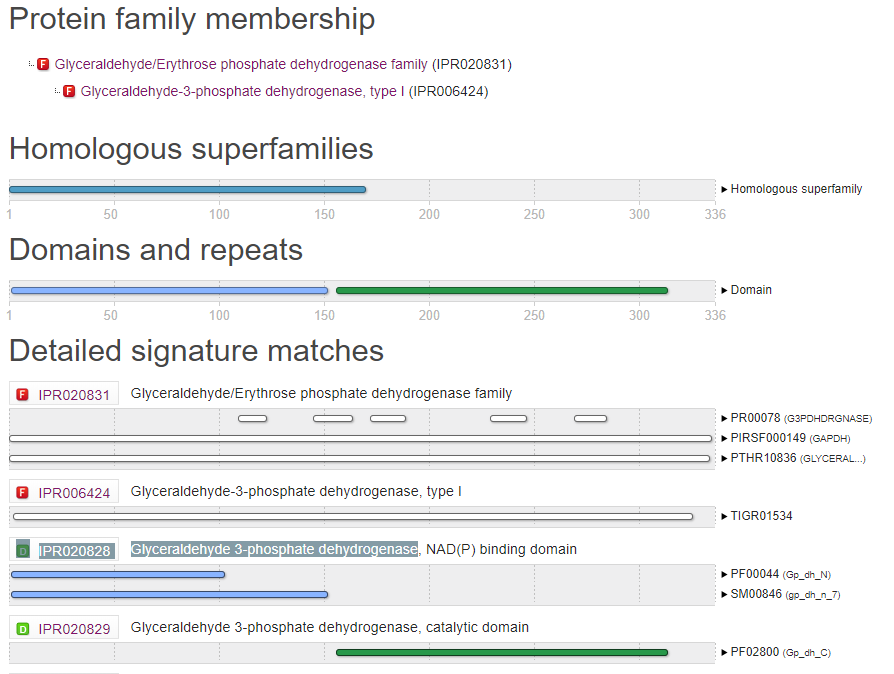


**Supplementary Figure 12**: Supplementary Figure: Scan result for ProteinGroup_16954 representative sequence. Sequence submitted 2/27/2018. Revealed domains IPR003953 and IPR015939 which represent an FAD binding domain and the C-terminal of Fumarate reductase/succinate dehydrogenase. The detailed signature match IPR011280 was linked specifically to succinate dehydrogenase/fumarate reductase flavoprotein subunit.

>P92|orf33424 COG1053 Succinate dehydrogenase/fumarate reductase, flavoprotein subunit

MIKIDSKIPEGPVAEKWTNYKAHQKLVNPANKRRLDIIVVGTGLAGASAAASLGEMGFRV

FNFCIQDSPRRAHSIAAQGGINAAKNYQNDGDSVYRLFYDTVKGGDYRAREANVYRLAEV

SNAIIDQCVAQGVPFAREYGGTLDNRSFGGAQVSRTFYAKGQTGQQLLLGAYSALSRQVN

VGTVKLYTRYEMQDVVIVDGRARGIIAKNLVTGELERFAAHAVVIATGGYGNAYFLSTNA

MGCNCTAAISCYRKGAVFANPAYVQIHPTCIPVHGDKQSKLTLMSESLRNDGRIWVPKKK

EDAVKLQKGEIKGSDIPEEDRDYYLERRYPAFGNLVPRDVASRAAKERCDAGFGVNNTGL

AVFLDFSEAINRLGIDVVLQRYGNLFDMYEEITDVNPGELAKEINGVKYYNPMMIYPAIH

YTMGGIWVDYELQTTIKGLFAIGECNFSDHGANRLGASALMQGLADGYFVLPYTIQNYLA

DQITVPRFSTDLPEFAEAEKAVQAKIDKFMSIQGKESVDSIHKKLGHVMWEYVGMGRTAE

GLKKGIAELKEIRKEFETNLFIPGSKEGMNVELDKAIRLYDFITMGELVAYDALNRNESC

GGHFREEYQTEEGEAKRDDENFFYVACWEYQGDDEKAPVLHKEPLVYEAIKVQTRNYKS


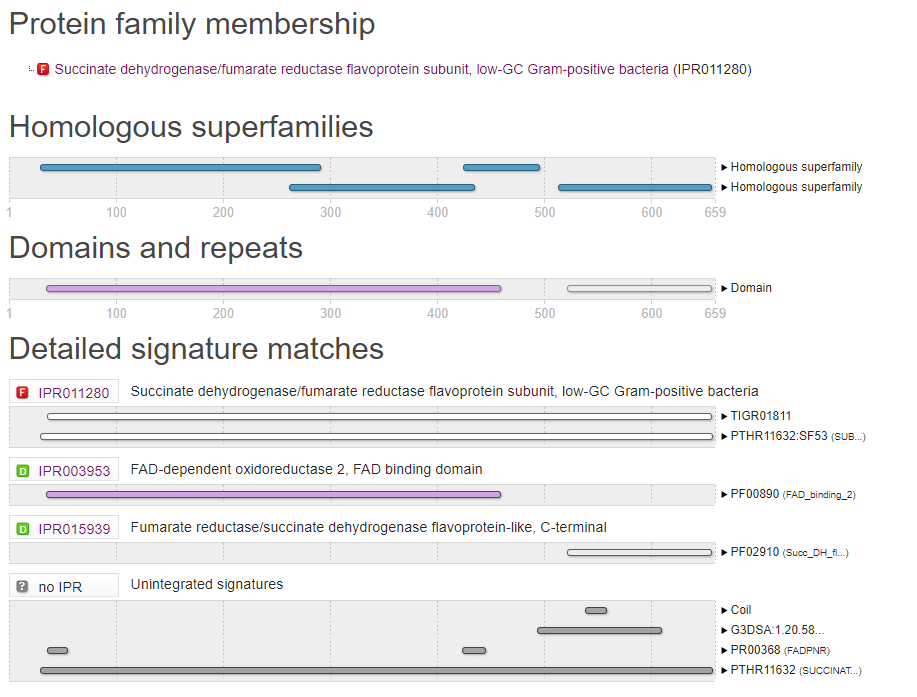


**Supplementary Figure 13**: Scan result for ProteinGroup_93252 representative sequence. Sequence submitted 2/27/2018. Revealed domain IPR000192 which is an aminotransferase class V domain. The detailed signature match IPR022278 was linked specifically to a phosphoserine aminotransferase.

>P58|orf66803 COG1932 Phosphoserine aminotransferase

MKKHNFNAGPSILPREVIEDTAKAILDFNGSGLSLMEISHRAKDFQPVVDEAEALFKELL

NIPEGYSVLFLGGGASMEFCMVPYNFLEKKAAYLNTGVWAKKAMKEAKGFGEVVEVASSA

EATYTYIPKDYTIPTDADYFHITTNNTIYGTELKKDLDSPVPMVADMSSDIFSRPIDVSK

YICIYGGAQKNLAPAGVTFVIVKNDALGKVSRYIPTMLNYQTHVDSGSMFNTPPVVPIYA

ALQTLRWIKAQGGVKEMERRAIEKADMLYAEIDRNKMFVGTAAKEDRSRMNICFVMAPEY

KDLEADFLKFATERGMVGIKGHRSVGGFRASCYNALPKESVQALIDCMQEFEKLH


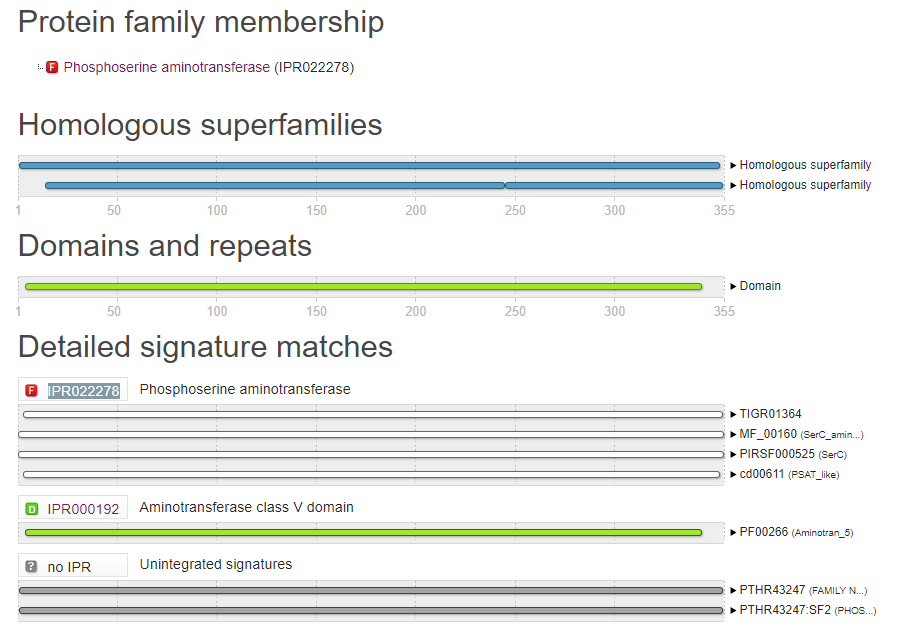


**Supplementary Figure 14**: Scan result for ProteinGroup_253057 representative sequence. Sequence submitted 2/27/2018. Revealed domains IPR031310 and IPR031309 which represent the N-Terminal and the C-Terminal respectively of an L5 ribosomal protein.

>P58|orf25533 COG0094 Ribosomal protein L5

MSNTASLKKEYAERIAPALKSQFQYSSSMQIPVLKKIVINQGLGMAVADKKIIEVAINEM

TTITGQKAVATISRKDIANFKLRKKMPIGVMVTLRRERMYEFLEKLVRVALPRIRDFKGI

ESKFDGKGNYTLGIQEQIIFPEINIDSITRILGMNITFVTSAQTDEEGYALLKEFGLPFK

NAKKD


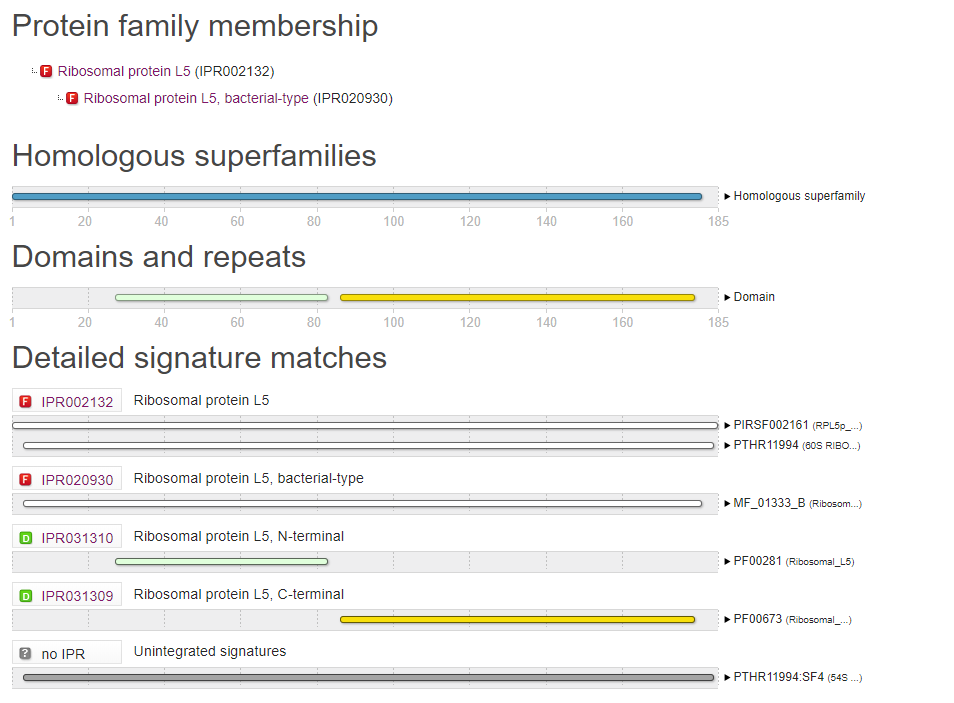


**Supplementary Figure 15**: Scan result for ProteinGroup_28742 representative sequence. Sequence submitted 2/28/2018. Revealed domain IPR019734 which is a Tetratricopeptide repeat which mediates protein-protein interactions. This domain, however, only captured a small percentage of the total sequence the rest of the sequence did not match any domains.

>P58|orf10751 NOG41185

MAMVVALFSSCSKKMGELSADYFTVTPQVLEAVGGKVPATINGKFPEKYFNKKAVVEVTP

VLKWNGGEAKGQPATFQGEKVEGNDQTISYKMGGSYTMKTSFDYVPEMAKSELYLEFKAT

IGKKVVTIPAVKIADGVISTSELVNNTLGNANPALGEDAFQRIIKEKHDANIMFLIQQAN

IRSSELKTAKEFNKEVANVNEAANKKISNIEVSAYASPDGGVSLNTTLAENREGNTTKML

SKDLKKAKIDAPIDAKYTAQDWEGFQELVSKSNIQDKELILRVIAMYQDPAQRESEIKNI

SAVYKELANTILPQLRRSRLTLNYEIIGKSDEEIAKLASSNPSELNVEELLYAATLTSDP

AKQEVIYTQATKQFPNDYRGYNNLGKLAYQAGNIDKAESYFKKAASVNATPEVNMNLGLI

SLMKGDKAAAEAYFGKAAGTKELGESMGNLYIAQGQYERAVNSFGDSKTNSAALAQILAK

DYNKAKNTLANVERPDAYTDYLMAVLGARTNNSSMVTSSLKSAVAKDSSLAKKAATDLEF

AKFFTNADFMNIIK


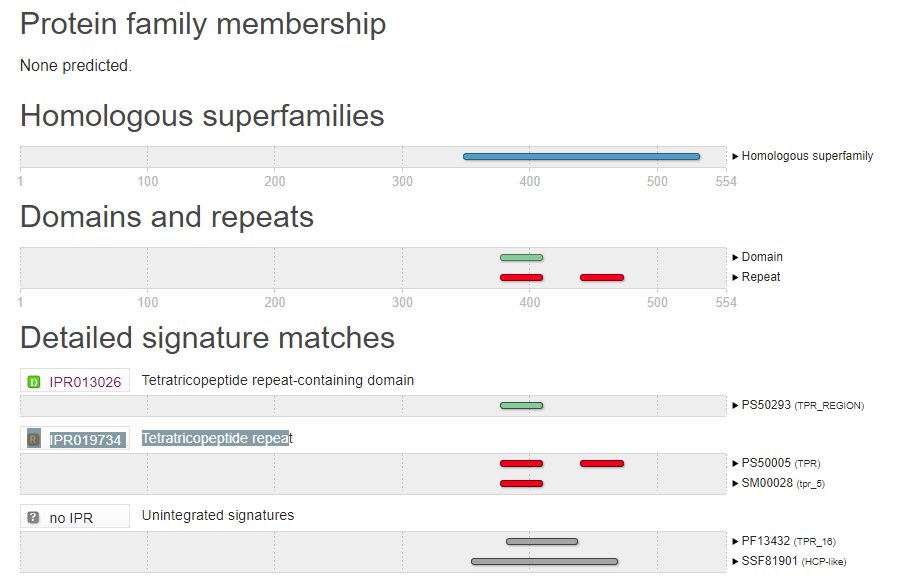


**Supplementary Figure 16**: Scan result for ProteinGroup_3177 representative sequence. Sequence submitted 2/28/2018. Revealed domains IPR012910 and IPR000531 which are a TonB-dependant receptor plug domain ,and TonB-dependant receptor-like beta barrel. Indicating that this may be a TonB dependent out membrane protein, but the specific function remains unknown.

>P92|orf38391 NOG238890

MNMEKCKYLLFCGTLALWMAGGFQQVYAATEPSSQAIQQQSNKVTGKVSDATGPIIGASV

VEKGTANGTITDLDGNFSLNVKAGATLVVSYVGYKSEEVKAGRGPLNITLKEDAKALDEV

VVTALGIKRERKALGYGIDEVKGEALTKAKETNLINSMAGRVPGLVVSQTAGGPSGSTRV

ILRGSTEMTGNNQPLYVVDGVPLDNTNFGSAGTNGGFDLGDGISSINADDVENMSVLKGP

AASALYGSRASHGVILITTKRANKDKISVEYNGTLTFDTQLAKWDEVQQIYGMGSNGTYS

YDAISNTNKSWGPKADGSNMLKYFDGVERPFLIVPDNTSNFFRTGITATNSAIIGVNSGK

TGIRFTYTDMRNKDIVPQTHMSRDIFNLRANTSAGKVDLDFSVNYTREDVKNRPALGDSK

SNIGKNLMTLATTYDQEWLQTYQTADGEYSNWNGMDPYNVNPYWDIYKNFNKSKKDLFRM

NGKAVWNIDPHLKLQATLGAELNWFTFDDYKAPTTPGFEAGRLQNSAFRNRMYNFEVLAL

YNNHWGDFDFNATLGGNVYKVNNQTTVTTAQDMKIRDVPSLTSFNEISVVPGSYRKQINS

VYGAVNVGWKHMLYLDATLRGDQSSTLPTGNNMYVYPSFSGSFVFSELTKLGDLLPYGKV

RMSWAQVGSDTDPYQLGLVYTKSKYAYPGYTIGYIDNGTIPNKDLKPTKTNSVEMGLELK

FLKNRIGLDFTYYSQISKNQIMGMASSWTTGYNYRLINAGKIENKGIEIALSTRPIQTRD

FSWDINLNFSKNSNKVKELDGESDMFELEKASWLDVQVAAKVGENFGSIVGPDFQRNEKG

DILIDPQTGLPQYDKSNHVLGNASWDWTGGLLTNFTYKNLSLVAVFDVKVGADLYSMSAR

AAYESGKSPETLAGRDAWYRSEEERLAAGIAKGADNWKPTGGFVAPGVIDNGDGTYRPND

IYINPEDYWMSVCRNAPSMFIYDNSYVKCRELTLSYNVPKSWLKNVVSGLTVSFVARNPF

IVWKNIPNIDPDSNYNNTTGMGLEYGSLPSRRSYGFNVNVKF


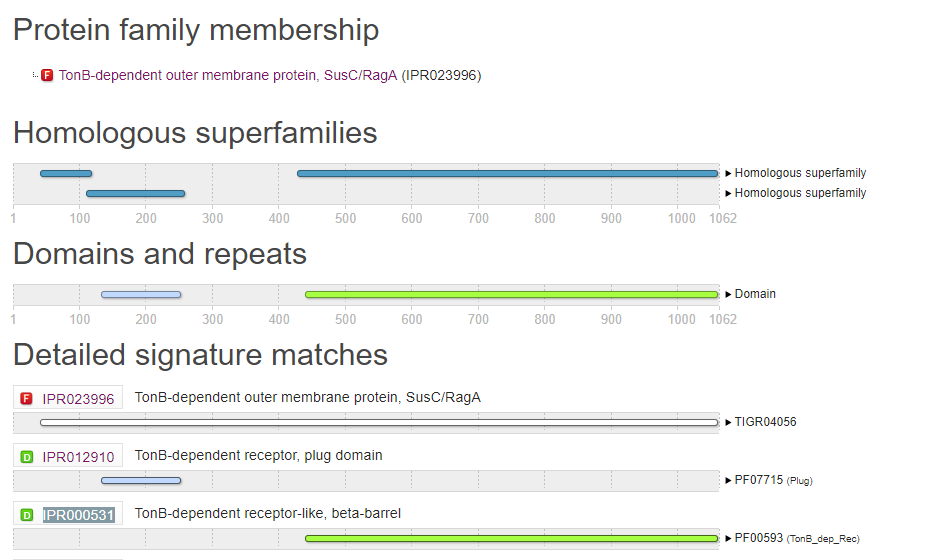


**Supplementary Figure 17**: Scan result for ProteinGroup_171203 representative sequence. Sequence submitted 2/28/2018. Revealed domain IPR002871 which is a Nitrogen Fixation Iron-sulfer cluster assembly cluster assembly, and which happens to be the NifU N-terminal domain.

>P104|orf74735 COG0822 NifU homolog involved in Fe-S cluster formation

MTYSHEVEHMCVVKKGPNHGPAPIPEEGKWVKAKEIVDISGLTHGIGWCAPQQGACKLTL

NVKEGVIQEALVETIGCSGMTHSAAMASEILPGKTILEALNTDLVCDAINTAMRELFLQI

VYGRTQSAFSEGGLMIGAGLEDLGKGLRSQVGTLYGTLAKGSRYLEMAEGYIKTIALDKN

DEICGYEFVHLGKFMDEIKKGTDANEALKKVTGTYGRFTEEQGAVKHIDPRHE


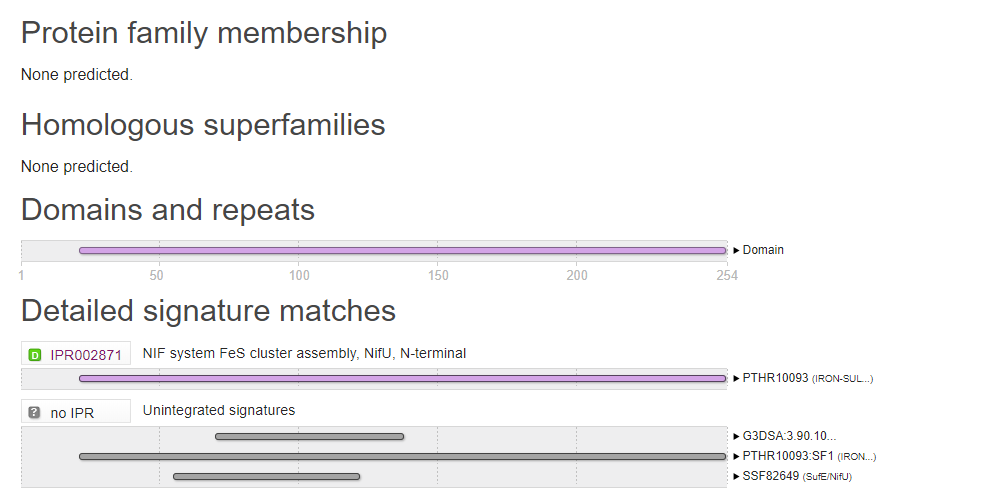


References

1. Pundir S, Martin MJ, O’Donovan C: **UniProt Protein Knowledgebase**. In: *Protein Bioinformatics: From Protein Modifications and Networks to Proteomics.* Edited by Wu CH, Arighi CN, Ross KE. New York, NY: Springer New York; 2017: 41-55.

2. The UniProt Consortium: **UniProt: the universal protein knowledgebase**. *Nucleic Acids Research* 2017, **45**(D1):D158-D169.

3. Finn RD, Attwood TK, Babbitt PC, Bateman A, Bork P, Bridge AJ, Chang H-Y, Dosztányi Z, El-Gebali S, Fraser M *et al*: **InterPro in 2017—beyond protein family and domain annotations**. *Nucleic Acids Research* 2017, **45**(D1):D190-D199.

4. Kanehisa M, Furumichi M, Tanabe M, Sato Y, Morishima K: **KEGG: new perspectives on genomes, pathways, diseases and drugs**. *Nucleic Acids Research* 2017, **45**(D1):D353-D361.

5. Cerveny L, Straskova A, Dankova V, Hartlova A, Ceckova M, Staud F, Stulik J: **Tetratricopeptide Repeat Motifs in the World of Bacterial Pathogens: Role in Virulence Mechanisms**. *Infection and Immunity* 2013, **81**(3):629-635.

6. Erickson AR, Cantarel BL, Lamendella R, Darzi Y, Mongodin EF, Pan C, Shah M, Halfvarson J, Tysk C, Henrissat B *et al*: **Integrated Metagenomics/Metaproteomics Reveals Human Host-Microbiota Signatures of Crohn's Disease**. *PLOS ONE* 2012, **7**(11):e49138.

7. Ouzounis C, Bork P, Sander C: **The modular structure of NifU proteins**. *Trends in Biochemical Sciences* 1994, **19**(5):199-200.

8. Kolmeder CA, de Been M, Nikkilä J, Ritamo I, Mättö J, Valmu L, Salojärvi J, Palva A, Salonen A, de Vos WM: **Comparative Metaproteomics and Diversity Analysis of Human Intestinal Microbiota Testifies for Its Temporal Stability and Expression of Core Functions**. *PLOS ONE* 2012, **7**(1):e29913.

9. Dem J, Curk T, Erjavec A, Group C, Hocevar T, Milutinovic M, Mozina M, Polajnar M, Toplak M, Staric A *et al*: **Orange: Data Mining Toolbox in Python**. *Journal of Machine Learning Research* 2013, **14**:2349-2353.
